# Computational analysis of auxin responsive elements in the *Arabidopsis thaliana L*. genome

**DOI:** 10.1186/1471-2164-15-S12-S4

**Published:** 2014-12-19

**Authors:** Victoria V Mironova, Nadezda A Omelyanchuk, Daniil S Wiebe, Victor G Levitsky

**Affiliations:** 1 Institute of Cytology and Genetics SB RAS, 10 Lavrentyeva avenue, Novosibirsk, 630090, Russia; 2 Novosibirsk State University, 2 Pirogova street, Novosibirsk, 630090, Russia

**Keywords:** Plant hormone, primary response, transcription regulation, composite element, bioinformatics

## Abstract

Auxin responsive elements (AuxRE) were found in upstream regions of target genes for ARFs (Auxin response factors). While Chip-seq data for most of ARFs are still unavailable, prediction of potential AuxRE is restricted by consensus models that detect too many false positive sites. Using sequence analysis of experimentally proven AuxREs, we revealed both an extended nucleotide context pattern for AuxRE itself and three distinct types of its coupling motifs (Y-patch, AuxRE-like, and ABRE-like), which together with AuxRE may form the composite elements. Computational analysis of the genome-wide distribution of the predicted AuxREs and their impact on auxin responsive gene expression allowed us to conclude that: (1) AuxREs are enriched around the transcription start site with the maximum density in 5'UTR; (2) AuxREs mediate auxin responsive up-regulation, not down-regulation. (3) Directly oriented single AuxREs and reverse multiple AuxREs are mostly associated with auxin responsiveness. In the composite AuxRE elements associated with auxin response, ABRE-like and Y-patch are 5'-flanking or overlapping AuxRE, whereas AuxRE-like motif is 3'-flanking. The specificity in location and orientation of the coupling elements suggests them as potential binding sites for ARFs partners.

## Background

The hormone auxin is a major regulator of plant growth and development. The influence of auxin on gene transcription is primarily mediated by its binding to TIR1/AFB receptors [1,2]. Auxin changes the conformation of receptors and thereby promotes their interaction with Aux/IAA proteins. These proteins form heterodimers with Auxin Response Transcription Factors (ARFs) making them inactive. TIR1/AFB receptors trigger ubiquitination of Aux/IAA proteins followed by their proteasome degradation. ARFs free from their co-repressors activate or repress transcription of their target genes. ARFs bind in target promoters to the specific sites called AuxREs (Auxin Response Elements) with the TGTCNN (most frequently TGTCTC) consensus core sequence [3-5]. Recently, it has been shown, that the TGTCGG motif is more effective in binding ARF1 and ARF5 [6].

About half of the *Arabidopsis thaliana *genes have at least one TGTCTC in any orientation within the first 1000 nt of their promoter regions [7]. Thus, TGTCTC consensus is really not a reliable method for AuxRE identification. Distribution and auxin-responsiveness of TGTCGG motifs in the plant genome have not been studied to date. While single TGTCTC hexamer does not confer auxin inducibility [8], this is provided by multimerized [4], or composite AuxREs [5]. Two copies of the TGTCTC element oriented as a palindrome or as a direct repeat (even if both sequences are inverse) are sufficient to provide auxin response [8,9]. ARFs bind as dimers on palindromic AuxREs where TGTCTC or TGTCGG are spaced by 5 to 9 nucleotides [3,6].

In the composite AuxREs, TGTCNC adjoins or overlaps with coupling (constitutive) elements [4,5]. For example, the coupling element CACGCAAT alone confers constitutive expression to a minimal promoter and the reporter gene shows no auxin response [5]. In functional composite AuxREs TGTCNC were found both in direct and reverse orientations [5,9,10].

Bioinformatic analysis of cis-elements in Arabidopsis and rice disclosed bZIP- and MYB-related binding sites as potential AuxRE coupling elements [11]. In this study, promoters (-1000 nt from TSS) of the auxin inducible genes in comparison to a randomized full genomic promoter dataset were enriched in AuxRE-, bZIP- and MYB-related elements and some of their composite modules in both genomes. The G-box (CACGTG), a type of bZIP-related motifs, binds BZR1 and PIF4 transcription factors [12,13], which recently were shown to heterodimerize with ARF6 [14]. ARF6 Chip-Seq analysis [14] revealed that composite AuxRE/G-box and AuxRE/HUD elements are highly enriched in ARF6 binding regions. The coupling motifs tend to be located close to the core AuxRE, mostly within 20 base pairs. There is also evidence of the functionality of AuxRE/MYB elements. First, the existence of ARF/MYB heterodimers was shown *in vitro *and *in vivo *for MYB77 and ARF7 transcription factors [15]. Second, auxin induction of seven well known auxin responsive genes having multiple putative MYB binding motifs in their promoters was greatly attenuated or abolished in both *myb77 *and *arf7 *knockout mutants. Third, the proximity of the MYB and AuxRE binding sites in one of them (IAA19) suggested that ARF7/MYB77 heterodimers may bind the composite elements in the promoter of certain auxin-responsive genes.

Also, there is evidence of other transcription factors forming heterodimers with ARFs. The BIGPETALp (BPEp) basic helix-loop-helix (bHLH) transcription factor and ARF8 interact through their C-terminal domains [16]. Double mutant analysis revealed synergic action of both transcription factors in limiting the first cell divisions during petal development and cell growth at later stages. The mutant defects were associated with changes in expression of the early auxin-responsive genes. Both transcription factors ARF5 and BES1, BZR1 homolog crucial for brassinosteroid response, bind to the SAUR15 promoter region containing composite AuxRE/HUD element [17].

Here we searched for the footprints of ARF dimerization with other transcription factors in the nucleotide context surrounding AuxRE. As a first approach, using information on experimentally proven AuxREs, we recognized potential AuxREs in the *Arabidopsis thaliana *genome. Together with the other AuxRE-related motifs [5,18] we analyzed their association with auxin-responsive expression. This allowed us to reveal some specificity in orientation and location of potential AuxREs in the auxin responsive promoters. Second, we performed a context analysis of the flanks in experimentally proven AuxREs and found three distinct types of potential coupling motifs (Y-patch, AuxRE-like, and ABRE-like). The analysis of a number of microarray datasets assured us that the composite elements with a specific orientation of AuxRE and the coupling motifs and the certain range of spacer length between them were associated with auxin responsiveness.

## Results

### Recognition of potential Auxin Responsive Elements

**Figure 1:**
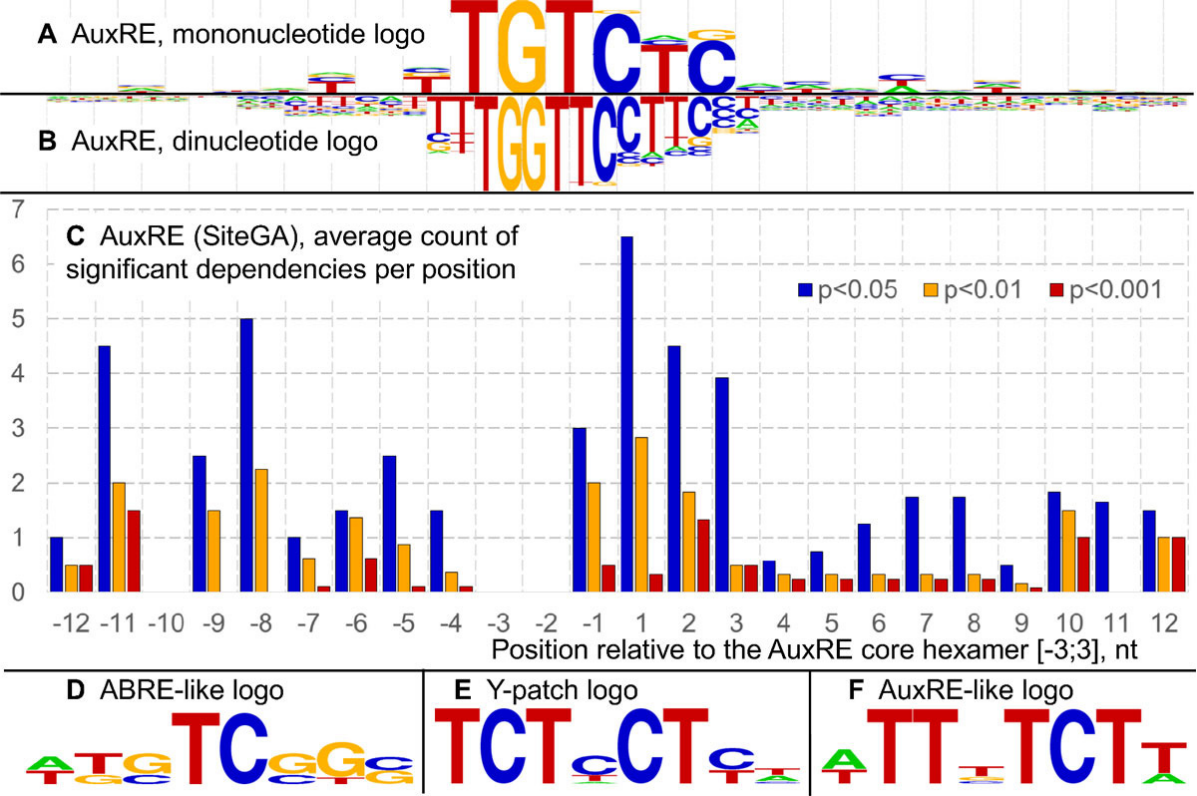
**Context pattern of AuxRE_P&S, _revealed by sequence analysis of experimentally proven AuxREs**. oPWM [19] provided for mono- (A.) and dinucleotide PWMs (B.). Logos for PWMs were computed according to [60]. C. SiteGA [19] revealed significant correlations between the frequencies of locally positioned dinucleotides. The average counts of significant dependencies are shown on the Y axis per positions along AuxRE site (X axis). AuxRE core TGTCNN locates in [-3;+3] nt. D - F. Logos for potential coupling elements found on the flanks of experimentally proven AuxREs. D. ABRE-like. E. Y-patch. F. AuxRE-like.

To apply methods for AuxRE recognition, we collected from published papers (a) the nucleotide sequences of experimentally proven AuxREs (*Training set*; 25 sequences 106-nt in length with centrally located TGTCNN hexamer, Additional file 1) and (b) extended promoters [-2000;-1] of auxin-regulated genes (*Positive set*; 44 sequences). We applied site recognition tools oPWM and SiteGA [19] to the *Training set *data. While the oPWM approach found some additional context features around TGTCNN core (Figure 1A-B, Additional file 2, Additional file 3), SiteGA revealed dependencies between locally positioned dinucleotides along the 24 nt AuxRE region (Figure 1C, Additional file 4). Next, we established the thresholds for genome-wide AuxRE recognition as those allowed to predict potential AuxREs in about 30% of promoters from the *Positive set *by both oPWM and SiteGA. Finally, potential AuxREs were predicted in the whole genome of *Arabidopsis thaliana *by: (a) oPWM/SiteGA combination (AuxRE_P&S_); (b) TGTSTSBC consensus [18]; (c) TGTCTC consensus [5], and (d) TGTCGG consensus [6].

To find potential coupling elements for AuxREs, we performed the motif search in the *Training set *using the MotiGA software (Additional file 5). MotiGA identified a number of motifs flanking TGTCNN core. Among the most overrepresented ones three main classes of similar motifs were distinguished (Additional file 3). We investigated further (see Section 4 of the results) one representative of each class: (a) pyrimidine-rich Y-patch, (b) ABRE-like motif, and (c) AuxRE-like motif (Figure 1D-F).

### Genome-wide statistics for potential AuxREs

**Figure 2:**
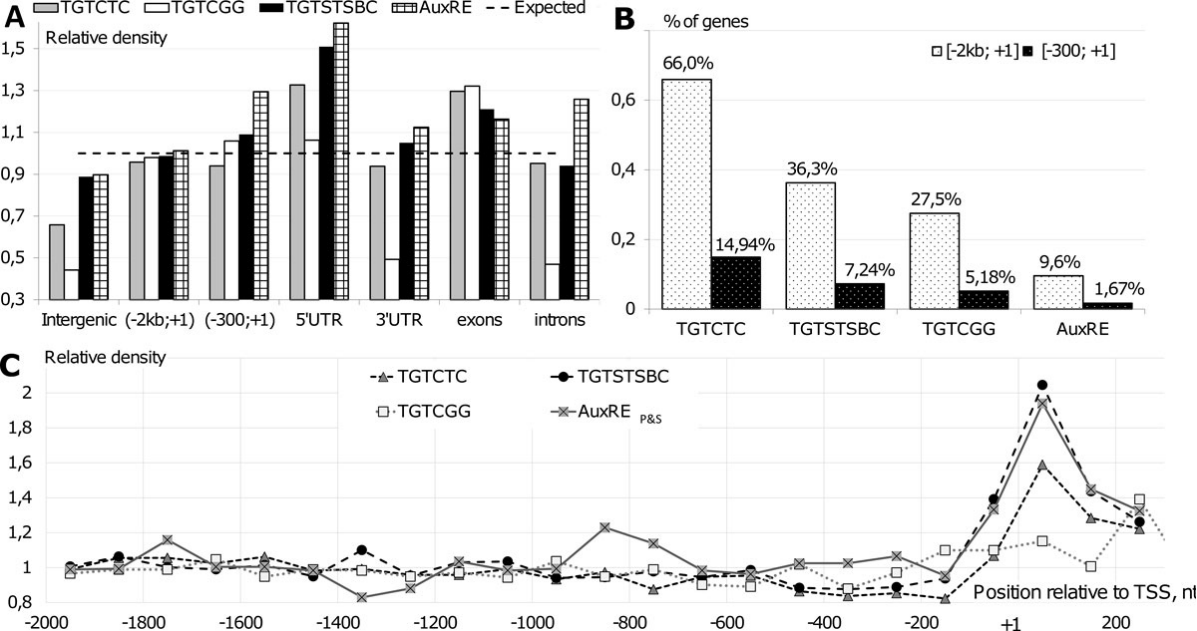
**Distribution of potential AuxREs in the *A. thaliana *genome**. A. The densities of four AuxRE variants (TGTCTC, TGTCGG, TGTSTSBC and AuxRE_P&S_) are shown in different genomic regions. Each density was normalized to an average density of the motif in the whole genome. B. The percentage of the Arabidopsis genes, having potential AuxREs in 2 kb and 300 bp upstream regions to the annotated TSS. C. Distribution of different AuxRE variants along the [-2000; +250] regions relative to annotated TSS. Each density on panels A and C was normalized to the average one of the respective AuxRE variant estimated for the whole genome.

We explored the abundances of four variants of potential AuxREs (TGTCTC, TGTCGG, TGTSTSBC and AuxRE_P&S_) in different genomic regions of *Arabidopsis thaliana*: intergenic and transcript (5'UTR, exons, introns and 3'UTR) (Figure 2A). Unexpectedly, all the AuxRE variants were found to be the most abundant in 5'UTR and exons. The density of AuxRE_P&S _were additionally enriched in introns, while TGTCGG was depleted there. TGTSTSBC, TGTCGG and AuxRE_P&S _have the above-average distribution density in proximal [-300; +1] promoters, AuxRE_P&S _was the most enriched among them.

The distribution densities of all the four AuxRE variants in the extended [-2000; +1] promoters were similar to the average in the genome (Figure 2A). The numbers of genes having these potential sites in the extended upstream regions were quite high: over 65% of genes contained TGTCTC, over 35% TGTSTSBC, 27.5% TGTCGG and about 10% AuxRE_P&S _(Figure 2B; Table 1). However, the distribution of all the four AuxRE variants along upstream regions is nonuniform, having a maximum around the Transcription Start Site (TSS) (Figure 2C). It has been shown that TGTSTSBC are enriched in the proximal [-250; +1] promoter of the auxin responsive genes [18]. Our results demonstrated that potential AuxREs are by more than 1.5 times dense around TSS than on average (except TGTCGG, which had lower density, but in a wider region). The regions of high distribution density spread both to the proximal promoter and 5'UTR. These results have an implication: 5'UTR should be considered in analysis of the auxin responsive regions.

**Table 1:** Genome-wide statistics for different AuxRE variants.

*AuxRE variant*	*Total amount of genes with AuxRE (Z)*	*Total amount of genes with conservative AuxRE*	*Up-regulated* *(1965 genes; 9.3%)^1^*				*Down-regulated* *(1916 genes; 9.1%)^1^*			
			
			With AuxRE		With conservative AuxRE		With AuxRE		With conservative AuxRE	
			
			*Amount (X_up_)*	*%, X_up_/Z^2^*	*Amount (Y_up_)*	*%, Y_up_/X_up_^3^*	*Amount (X_down_)*	*% , X_down_/Z^2^*	*Amount (Y_down_)*	*% , Y_down_/X_down_^3^*
**TGTCTC**	12635	4496	1221	9.7%	927	75.9%	1141	9.0%	746	65.4%
**TGTCGG**	5054	1696	449	8.88%	334	74.4%	454	8.98%	297	65.42%
**TGTSTSBC**	6829	1978	657	9.6%	504	76.7%*	605	8.9%	396	65.5%
**AuxRE_PWM_**	3799	1020	372	9.8%	290	78.0%*	354	9.3%	229	64.7%
**AuxRE_SGA_**	4679	1321	481	10.3%*	364	75.7%	438	9.4%	296	67.6%
**AuxRE_P&S_**	1779	468	191	10.7%*	158	82.7%**	167	**9.4%**	115	**68.9%**

For the genes with predicted AuxRE in the [-1500; 5'UTR] region, the total amount and the amount of auxin-regulated genes are given. The AuxRE considered as conserved if its TGTC core could be found in an alignment of the regulatory region among *A. thaliana, A. lyrata, T. halophile, C. papaya and C. clementine *(See Materials and Methods). The set of auxin regulated genes created by meta-analysis of microarray data (Table 1) contains 1965 up- and 1916 down-regulated genes, which significantly (more than 1,5-fold, p < 0,05) changed their expression relative to control in more than three microarrays. The portion of auxin-regulated genes among the genes having an AuxRE variant was compared with an average portion of auxin responsive genes in the whole genome using the t-test for proportions (see Materials and Methods). The highest portions are shown in bold.

^1^Total number of auxin regulated genes and its portion from the whole-genome set (21098 microarray probes)

^2^The portion of auxin regulated among the genes having an AuxRE variant, compared with an average portion (^1^) by the t-test for proportions (*p < 0,05).

^3^Relation between conservation of an AuxRE variant in a gene promoter and its auxin responsiveness. To estimate the of statistical significance, the *Y_up_/X_up _*portion was compared by the t-test for proportions with 1442/1965 portion, as 1442 out of 1965 up-regulated genes contained any conserved position in a multiple alignment of the regulatory region. *Y_down_/X_down _*was compared with 1217/1916 portion (*p < 0,05; ** < 0,005).

### AuxRE_P&S _predicts auxin-regulated genes better than other models

To test which AuxRE model predicts better auxin responsive elements, we performed meta-analysis of publicly available microarray experiments with auxin treatments (16 microarrays, Table 2). First, we created a list of auxin-regulated genes which significantly changed their expression (by more than 1.5-fold, p < 0.05) in over three microarrays experiments. The threshold for the number of microarrays was set by the binomial trial estimate (see Methods). The resulting list contained 1965 up-regulated and 1916 down-regulated genes. Second, the fractions of the significantly up- or down-regulated genes with an AuxRE variant in their promoter and 5'UTR regions were compared with that for all the genes tested in the experiment (Table 1, Additional file 6). The statistical significance of the difference between the fractions was estimated by the t-test for proportions (See Methods). The analysis showed that the targets with AuxREs predicted by simple models (TGTCTC and TGTSTSBC and AuxRE_PWM_) were not enriched in the auxin-regulated genes, whereas those predicted by more complex models (AuxRE_SGA _and AuxRE_P&S_) were significantly enriched (p < 0.05). Notably, the fraction of the auxin up-regulated genes among those with predicted AuxRE_P&S _(10.7%) was higher than the respective fractions for AuxRE_SGA _(10.3%) and AuxRE_PWM _(9.8%), which substantiates that usage of two models based on different principles improves prediction (Table 1). No statistical significance was found for the down-regulated by auxin genes.

**Table 2:** Microarray experiments analyzed in this paper for association between the presence of potential AuxRE and auxin responsive gene expression

*N*	*Microarray ID*	*Treatment*	*Tissue*	*References*
1	GSE627	5 µM IAA, 2 h	7 dag seedling	[36,64]
2 3 4 5	GDS672	0.1 µM IAA, 1 h 1 µM IAA, 1 h 0.1 µM IAA, 3 h 1 µM IAA, 3 h	10 dag seedling	[65]
6	GDS3505	1 µM IAA, 4 h	Roots of 3 dag seedlings	[66]
7	GDS1408	10 µM IAA, 30 min	Flower	[67]
8 9	GDS1515	10 µM NAA, 2 h 10 µM NAA, 6 h	Root segments of 3 dag seedlings	[68]
10	GDS1044	10 µM IAA, 1 hr	7 dag seedling	[69]
11	GDS744	10 µM IAA, 2 hr	5 dag seedling	[41]
12 13 14 15 16	GSE35580	5 µM IAA, 2 hr	7 dag seedling, roots Root, epidermis Root, pericycle Root, stele Root, columella	[70]

The applicability of different models for AuxRE recognition was also tested using the data on phylogenetic footprinting (See Methods). For the analysis we used the data from Vista tool [20] on the whole genome alignments between four plant species. We tested how many potential AuxREs were conserved, namely located in an alignment with a high score and had no substitutions in the TGTC core (See Methods). About one third of AuxRE variants were found to be conserved for each of the AuxRE variant. Then, we analyzed how many of the auxin-regulated genes with a potential AuxRE obtained a conserved one (Table 1, Additional file 6). The portions were again the highest for AuxRE_P&S _(82.7% of the up-regulated genes and 68.7% of the down-regulated). The difference in comparison with an average conservation in the regulatory regions (See Methods) was significant only for the up-regulated genes with conserved TGTSTSBC (76.7%, p < 0.05), AuxRE_PWM _(78%, p < 0.05) and AuxRE_P&S _(82.7%, p < 0.005).

The analysis provided proof that, indeed, AuxRE_P&S _predicts auxin responsive elements better than the other existing models.

### The preferences in location and orientation of potential AuxREs associated with auxin response

A more detailed meta-analysis of microarray data was performed to analyze the associations between the presence of AuxRE_P&S _in a gene promoter (Additional file 7) and its auxin responsiveness. Independently of each of the 16 microarrays (Table 2), we tested the statistical significance between the fractions of the up- or down-regulated genes having and not having AuxRE_P&S _(See Methods). The association between the presence of a potential AuxRE and auxin-responsive gene expression was considered as significant, if it was revealed in more than three microarrays, the threshold was set by the binomial trial estimate (See Methods). A significantly higher portion of auxin upregulated genes was found in five and seven microarrays for the genes with AuxRE_P&S _predicted in the extended and proximal upstream regions, respectively (Table 3; Additional file 8). No significant association was found between the presence of a single AuxRE_P&S _and auxin down-regulation. Interestingly, the significant associations were found mainly in the microarrays, where an early auxin response (2 h treatment and less) was investigated (Additional file 8). No association with auxin dosage was found.

**Table 3:** Summary for associations between the presence of AuxRE_P&__S _and auxin response detected by statistical analysis of 16 microarray experiments (Table 2).

*Up- or down-regulation*		*up*	*down*	*up*	*down*
Number, orientation and location of AuxRE_P&S_		[-300;5'UTR]		[-1500;5'UTR]	

Single	AuxRE_P&S_	7*	3	5*	1
	AuxRE_P&S_+	4*		4*	
	AuxRE_P&S_−	3		3	

Multiple	AuxRE_P&S_		2	7*	1
	AuxRE_P&S_+		2	1	3
	AuxRE_P&S_−		2	6*	1

For each microarray experiment, we estimated, if the difference in portions of auxin regulated genes (>1.5-fold change, *p *< 0.05) between the subsets of gene having specifically localized and/or oriented AuxRE_P&__S _and whole set of genes tested was significant in the microarray. In the cells, we present the number of microarrays in which the significant difference was found, empty cells mean no difference. The threshold at which the association between the presence of AuxRE_P&__S _and auxin responsive expression was considered as significant was set as three microarrays (see Methods). (+) means direct orientation; (−) - reverse orientation of the AuxRE_P&S _elements.

*Significant associations

Notably stronger association with auxin upregulation was found for directly oriented single AuxRE_P&S _than for reverse ones (Table 3). However, among the genes with multiple AuxRE_P&S, _significant association was found for reverse AuxRE_P&S_, rather than direct AuxRE_P&S_.

The same analysis for TGTCTC, TGTCGG and TGTSTSBC confirmed the results described above for AuxRE_P&S _(Additional file 8) and allowed us to conclude that functional AuxREs: (1) tend to be located in proximal promoters; (2) mediate transcriptional activation in response to auxin; (3) single AuxREs preferably have direct orientation, while multiple AuxREs are reversely oriented.

### Functional annotation of composite auxin responsive elements

We found three types of the coupling motifs (Figure 1D-G) on the flanks of experimentally proven AuxREs. Pyrimidine-rich TCTCCTYT motif (Figure 1D) in its sequence and distribution resembles Y-patch [21] or TCT-motifs [22], both related to transcription initiation. We designate further this coupling motif as Y-patch. HDSDYSKS motif (Figure 1F) was found as significantly matching with the EmBP-1 binding site (Additional file 9) by the TomTom tool [23]. It was shown that the transcription factor Embp-1 interacts with the ABA-responsive element (ABRE) in the 5'-regulatory region of a wheat gene [24], which is why we further refer to HDSDYSKS as ABRE-like. The third type of AuxRE-flanking motif DTTBTCTH meets AuxRE itself (Figure 1G). Since B=T/G/C and H=T,G,C, a third part of the AuxRE/AuxRE-like composite elements recognized genome-wide have two TGTCNN core motifs. As AuxRE repeats were identified in promoters of the first analyzed auxin-responsive genes [5,25], this was the expected result.

We recognized three types of composite AuxREs in *A. thaliana *extended upstream regions and analyzed (a) their distribution relative to TSS; (b) the functional annotation of the genes having different composite elements in their upstream regions in the AgriGO database [26]; (c) their impact on auxin responsive gene expression.

#### AuxRE/Y-patch

**Figure 3:**
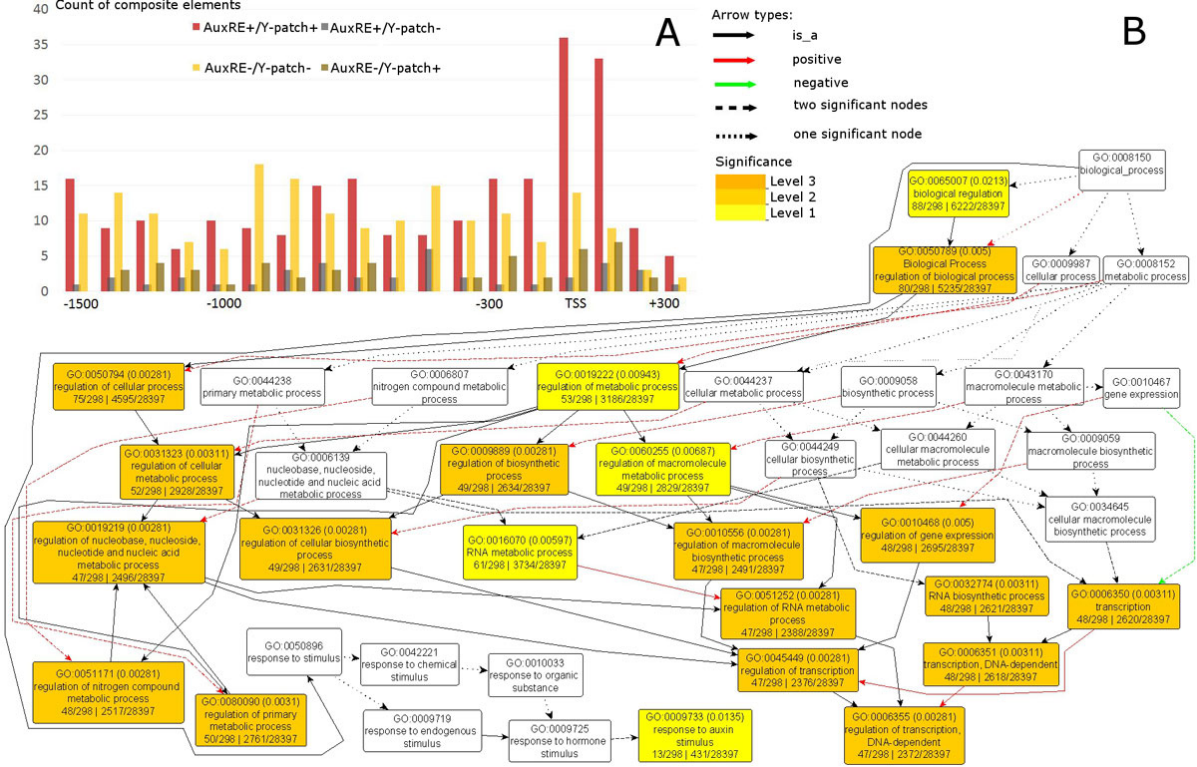
**Distribution and functional annotation of the predicted composite AuxREs/Y-patch**. Panel A depicts the distribution of potential composite AuxREs relative to TSS (position +1). Panel B shows the functional annotation (GO) of the genes having the composite element AuxRE+/Y-patch+ in their regulatory regions. Functional annotation was performed using AgriGO analysis tool [26]. Annotation is represented as a graph in which each node represents a definite GO term with the value of FDR in brackets. The color of the node, from yellow to deep orange reflects the level of significance of the term which is represented by a given node. Each node is connected with the other by edges of different structure and color (annotation is given).

**Figure 4:**
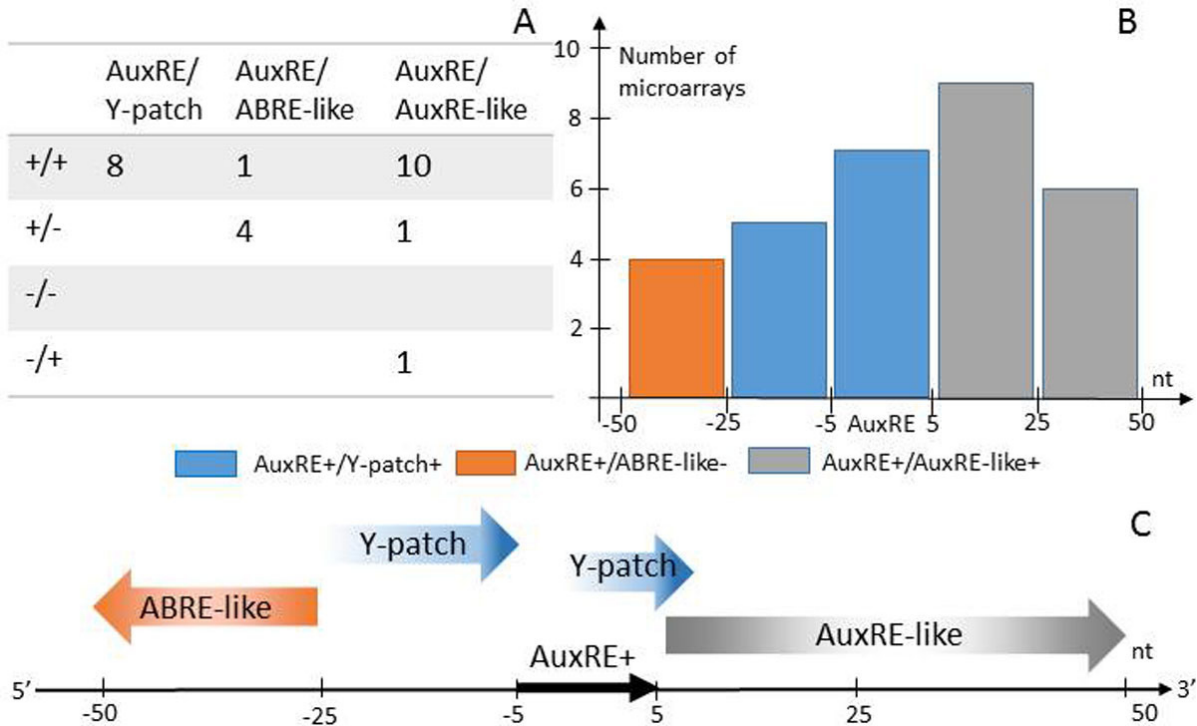
**The preferences in the orientation (A) and the location (B-C) of the coupling motifs around AuxRE_P&S _in the composite auxin responsive elements**. A. The number of microarray experiments where the composite elements with the specific orientation of the coupling motifs were found significantly associated with auxin responsiveness (See Methods). B. The significant associations found by microarray data analysis for AuxRE+/Y-patch+, AuxRE+/ABRE-like- and AuxRE+/AuxRE-like+ composite elements, depending on the positioning of the coupling motif relative to AuxRE. The distance between the centers of the coupling motif and anchor AuxRE in *nt *is plotted along the x axis. The core AuxRE sequence TGTCNN locates from -3 to +3 nt. C. The conceptual summary about the composite structure of auxin responsive elements.

Composite AuxRE/Y-patch elements are asymmetrically distributed around TSS, having a maximum close to TSS with a shift to 5'UTR (Figure 3A). This distribution is a characteristic feature of the composite elements where both AuxRE_P&S _and Y-patch were identified in the direct orientation (AuxRE+/Y-patch+). Genes having AuxRE+/Y-patch+ where functionally annotated were significantly (p < 0.002, Hochberg FDR test) related to auxin response, regulation of metabolic and cellular processes, regulation of transcription (Figure 3B). Moreover, in half of tested microarrays AuxRE+/Y-patch+ composite elements were significantly associated with auxin up-regulation (Figure 4A).

#### ABRE-like motif

Composite element AuxRE/ABRE-like as AuxRE/Y-patch is also enriched around TSS, and the elements with the AuxRE in the direct orientation (AuxRE+/ABRE-like+ and AuxRE+/ABRE-like-) impact on this asymmetry (Additional file 10). Functional annotation and microarray data analysis revealed enrichments for AuxRE+/ABRE-like elements (Additional file 11; Additional file 9). In microarray data meta-analysis, AuxRE+/ABRE-like-elements were the most attributed to auxin response among AuxRE+/ABRE-like elements (Figure 4A; Additional file 9). Functional annotation of the genes having AuxRE+/ABRE-like composite elements also revealed their significant enrichment (p < 0.002, Hochberg FDR test) in the genes involved in regulation of various biological processes, such as development, regulation of transcription and response to external stimulus (Additional file 11).

#### AuxRE-like

AuxRE/AuxRE-like with direct orientation of both coupling motifs are enriched around TSS as the other two composite elements are (Additional file 10). In the analysis of microarray data, AuxRE+/AuxRE-like+ composite elements showed the best results, revealing significant associations with auxin upregulated genes in ten out of sixteen microarray experiments (Figure 4A). The functional annotation of the genes with predicted AuxRE+/AuxRE-like+ elements in their upstream regions also revealed the significant relation (p < 0.002, Hochberg FDR test) with growth and developmental processes (Additional file 12). Based on this analysis, AuxRE+/AuxRE-like+ were also found associated with shoot morphogenesis.

### An important role of a spacer in the composite elements

The above analysis based on microarray data did not take into account the length of the spacer between potential AuxRE and the coupling motif. To do this, we considered six locations for the coupling motifs relative to the center of potential AuxRE either in 5' or 3' directions: overlapped (5 nt between the centers or less) and with short (from 6 to 25 nt) or long (from 26 to 50 nt) spacers (Figure 4). The subsets of genes carrying the composite element with the specific orientation and location of the coupling motif relative to AuxRE_P&S _were analyzed separately. The composite element variant was considered as influential, if it was found significantly associated with auxin response in at least four microarrays (see Methods). In Figures 4B-C only the influential composite elements are shown. Three types of composite AuxRE_P&S _had different preferences in the coupling motif positioning, but they all had directly oriented core AuxRE_P&S _(Figure 4C). AuxRE-like motif tends to be located also directly on the 3'flank with no preferences to the length of the spacer. Y-patch needs to be located closely. We found two different types of AuxRE/Y-patch elements: Y-patch overlapping AuxRE core sequence at the 3' side and Y-patch located 5' to the core with a short spacer. ABRE-like motifs are located on the 5' flank of AuxRE with a long spacer.

## Discussion

The auxin signaling pathway has multiple players with diverse interactions. In Arabidopsis, the ARF family of transcription factors consists of 23 members, forming heterodimers with 29 Aux/IAA co-repressors [27]. ARFs also interact with other transcriptional co-regulators like TOPLESS [28], SEUSS [29] and with other transcription factors like MYB77 [15] and BPEp [16]. The diversity in the interaction map for ARF transcription factors may be in some way footprinted in the regulatory regions of auxin-responsive genes, for example, as composite AuxRE elements. Indeed, ARF homodimers need two closely located binding sites with a spacer of a certain length [30].

Here we performed sequence analysis of the experimentally proven AuxREs with their flanks (Additional file 1) to reveal: (1) a nucleotide context of AuxRE itself; (2) coupling motifs, which together with AuxRE may form composite elements.

### In search of new AuxRE variants

There were three AuxRE variants revealed to date - TGTCTC [5], TGTSTSBC [18] and TGTCGG [6]. Distribution of TGTCGG in regulatory regions and its relation to auxin response has not been studied to date. The TGTCTC motif is present in upstream regions of approximately 65% of the *A. thaliana *genes (Figure 2B), which makes it over predictive for AuxRE. TGTSTSBC is less sensitive in recognizing AuxRE, but more specific than the canonical TGTCTC [18]. Despite the gain in specificity, the number of potential AuxRE identified by TGTSTSBC consensus was still too high (Figure 2B). However, not all early auxin responsive genes have any of these two AuxRE variants in their proximal promoters. If we still suppose that auxin response of these genes is mediated by ARFs, we should expect either the existence of other AuxRE variants or another location for these AuxREs.

To search for new AuxRE variants, we collected sequences of all AuxREs experimentally confirmed to date (Additional file 1). Application of two recognition models, oPWM and SiteGA, that are based on different approaches [19], allowed us to reveal context features of AuxRE (Figure 1; Additional file 2, Additional file 3, Additional file 4) and to deduce the new variant for AuxRE (AuxRE_P&S_). The genes having AuxRE_P&S _in their extended promoters (Additional file 7) only partly overlap with those having TGTCTC and/or TGTSTSBC in the same region (data not shown). Microarray data analysis showed that the presence of AuxRE_P&S _in the extended promoters correlates with auxin up-regulation better than those for TGTSTSBC, TGTCGG or TGTCTC (Additional file 8). All the four investigated AuxRE types showed good correlations with auxin up-regulation when they located in the proximal promoters.

We also tested the relation between conservation of an AuxRE variant in a gene promoter and its auxin responsiveness. As a result, we established that among the auxin up-regulated genes with potential AuxRE_P&S _the highest gene fraction was obtained for the conserved ones (Table 1, Additional file 6). These genes maintain the core TGTC sequence in a conserved regulatory region among four plant species: *A. thaliana, A. lyrata, T. halophile, C. papaya, and C. clementine*. Among the auxin up-regulated genes of the other AuxRE variants there were lower fractions of conserved sites.

### AuxREs mediate auxin responsive up-regulation

Extensive analysis of association between auxin response and the presences of each AuxRE type in the regulatory regions of various lengths allowed us to conclude that AuxREs mainly regulate auxin responsive activation of transcription. The influence of AuxREs on gene down-regulation is expected to be minor, as among thousands of tested AuxRE types (different variants of AuxREs, single/multiple AuxREs, various composite elements) none was found significantly associated with down-regulation (Additional file 9). This finding well agrees with the previously published analysis of auxin responsive transcriptome [31], where it was shown that the primary auxin response was almost entirely restricted to up-regulated genes. Also, the prevalence of AuxRE-dependent transcriptional activation in primary auxin response came from the analysis of Aux/IAA-ARF interactome, which revealed that sensitive to auxin Aux/IAA proteins mainly form dimers with ARF activators [32].

### Functional AuxREs appeared to be enriched around TSS

We showed here that potential AuxREs are not uniformly distributed in the Arabidopsis genome with the highest density in 5'UTRs (Figure 2A). Aligning the AuxREs densities along the promoter and 5'UTR regions, we found that all the three AuxRE composite elements are enriched around TSS (Figure 2C). It has been demonstrated earlier [18] that AuxRE are enriched in proximal promoters of the auxin regulated genes. We confirmed this statement and extended it to the gene regions located downstream TSS. Two following independent results of our computational analysis support the idea that functional AuxREs appeared to be enriched around TSS.

First, microarray data analysis provides an insight that there is a strong association between the presence of potential AuxREs in the proximal promoter and 5'UTR and auxin responsiveness. Analyzing auxin responsive expression of the genes having predicted AuxRE_P&S_, TGTCGG, TGTCTC or TGTSTSBC in [-300; 5'UTR] regions, we found among them a significantly higher portion of auxin up-regulated genes (comparing with the whole microarray gene set) in more than seven of the tested sixteen microarrays (Additional file 8). For the extended [-1500 bp; 5'UTR] regions, the significant differences were revealed in fewer microarrays: five for AuxRE_P&S _(Table 1, Additional file 6), TGTCGG and TGTCTC and three for TGTSTSBC.

Second, all the three types of composite AuxRE elements were enriched around TSS (Figure 3). The composite elements accumulated around TSS, namely AuxRE+/Y-patch+, AuxRE+/ABRE-like and AuxRE+/AuxRE-like+, showed significant association with auxin response in microarray data analysis (Figure 4A). Functional annotation of the genes having the AuxRE+/Y-patch+ elements also showed significant enrichment of the genes related to response to auxin stimulus (Figure 3B). Functional annotation of genes carried out for other enriched around TSS composite elements (AuxRE+/AuxRE-like+, AuxRE+/ABRE-like) also showed significant results for developmental and regulatory processes (Additional file 11, Additional file 12).

Enrichment of AuxREs in 5'UTR has not been revealed earlier. However, in some auxin responsive genes potential AuxRE were described in 5'UTR. Composite AuxRE elements (AuxRE in the close vicinity to MYB binding sites) were found in 5'UTR of the IAA19 gene [15]. ChIP analysis revealed potential AuxRE in 5'UTR of the TMO5 gene [33]. There are two explanations of this bias for potential AuxREs towards downstream regulatory regions. The first is poor annotation of TSS in the *A. thaliana *genome or the existence of multiple and alternative TSS sites. The second relies on the mechanism of primary auxin response, which probably needs binding of ARF transcription factors in the close proximity to RNA polymerase complex, without specificity in location relative to TSS. In animals, there are some examples of transcription factors, whose binding sites distribution spreads below TSS, for example p53 [34] and GAGA [35]. Hence, it may be assumed that the distribution of some transcription factor binding sites around TSS may be a fundamental feature of the eukaryotic transcription initiation machinery.

### Orientation of AuxRE influences auxin response

As there was no difference in auxin responsiveness of DR5 reporter versions with direct or reverse repeats of CCTTTTGTCTC motif [8], it has been proposed that there is no specificity in orientation of all AuxRE elements [9]. And, indeed, reversely oriented AuxREs were experimentally proven in several auxin responsive genes, for example AtLBD29 [36] and OsCRL1 [37].

However, by genome-wide analysis of associations between the presence of potential AuxRE_P&S _and auxin responsive gene expression, we found significant difference for direct and reverse AuxRE_P&S_. Single AuxRE_P&S _significantly associated with up-regulation were directly oriented, while multiple AuxRE_P&S _were reversed (Table 1, Additional file 6). All the composite elements, which were found associated with auxin response, had direct AuxRE_P&S _core (Figure 4, Additional file 8).

### Composite Auxin Responsive Elements

A composite element contains two functionally linked binding sites for distinct transcription factors and in this way provides the basis for integrating the inputs of two signaling pathways and enabling specificity of expression [38]. In the simplest case, the composite element corresponds to a pair of individual binding motifs located at a particular distance from each other and involved in formation of specific DNA-protein-protein-DNA complexes [39]. In the COMPEL database, several hundred composite elements are described with evidences that their regulatory functions differ from those provided by their individual components [40]. As ARF transcription factors are known to form homo- and hetero-dimers, by sequence analysis of experimentally proven AuxREs we expected to find several AuxRE coupling motifs. Unlike the previous study of coupled regulatory motifs in promoters of auxin responsive genes [11,41], we searched only for the composite elements with closely located coupling motifs (within 50 nt). As a result, we found and analyzed three distinct types of composite elements: AuxRE/Y-patch, AuxRE/ABRE-like and AuxRE/AuxRE-like (Figure 1D). Using microarray data analysis and functional annotation, we showed that among the genes having the composite elements in their upstream regions there were significantly higher portions of auxin regulated genes in comparison with those having a single potential AuxRE (Additional file 8).

#### AuxRE/ABRE-like composite element

The first discovered composite AuxRE named D1-4 consists of 11 nucleotides CCTCGTGTCTC, where 3'located TGTCTC overlaps by 3 nucleotides with 5'located reverse oriented ABA response element, ABRE [5]. ABRE elements contain G-box (ACGA), which is bound by bZIP transcription factor EmBP-1 [24]. EmBP-1 homologs specifically bind auxin-responsive D1-4 [5]. In this work we encountered again on AuxRE/ABRE-like composite elements. The degenerative ABRE-like motif was discovered on the flanks of experimentally proven AuxREs in the regulatory regions of *GH3, BRX, TMO7, IPT5 *and *DRN A. thaliana *genes (Additional file 2). Functional annotation and expression level analysis for the genes with AuxRE/ABRE-like composite elements in the promoter and 5'UTR regions allowed us to specify the structure of functional AuxRE/ABRE-like. The composite element having the major impact on auxin responsiveness consists of direct AuxRE_P&S _with 5' flanking ABRE-like with a long spacer (Figure 4B-C). In three microarrays, we also found a significant association of auxin response with the presence of the reversed AuxREs overlapped from the 5' side with reversed ABRE-like motif (Additional file 8). Despite, the result was insignificant, it appeared of interest, because the latter AuxRE/ABRE-like type resembles D1-4 composite element [5].

#### AuxRE/Y-patch composite element

The second AuxRE coupling motif recognized on the flanks of experimentally proven AuxREs was CT-rich motif similar to Y-patch (Additional file 3). Y-patch sequence was found significantly enriched around TSS in different plant genomes [21,42]. Whereas TATA box is located within −45 to −18 in 25% of *A.thaliana *promoters, Y-Patch is located around TSS (from −50 to +50) in 49% of genes [42], which suggested their important role in transcription initiation [21]. Here we found significant association between the presence of AuxRE/Y-patch composite element in gene upstream regions and its auxin responsiveness (Additional file 8).

The composite elements with the direct orientations of the coupling AuxRE and Y-patch were found significant by functional annotation and microarray data analysis (Figure 3A-B; 4A; Additional file 8). Both AuxRE and Y-patch were enriched around TSS, so the composite AuxRE/Y-patch might be just a consequence of their co-localization. However, the analysis of the relative positions and orientations of the AuxRE/Y-patch coupling elements suggests that they are not just located in the same region, but also need a specific structure, namely directly oriented Y-patch overlapped with the direct AuxRE on its 3' flank or located 3' with a short spacer (Figure 4). All the other combinatorial variants were found to be irrelevant to the auxin response.

Y-patch functionality has not been revealed yet [21], our data provide an idea that a protein, which binds to Y-patch, may interact with an ARF transcription factor and impact thus on auxin response.

#### AuxRE/AuxRE-like composite element

In auxin responsive gene promoters one usually may find multiple copies of potential AuxREs. The multimerized AuxREs were experimentally shown as providing auxin responsive gene expression [9]. For example, the promoter of the widely used auxin sensor DR5 consists of 7 AuxREs [8]. Recently, it has been shown that AuxRE-AuxRE reverted repeats are bound by ARF homodimers with a spacer-sensitive specificity [6]. According to [41], 42% of auxin responsive promoters contain at least one pair of TGTC sites within 50 bp. Analysis of auxin responsive regions with multiple AuxREs suggested that they cooperate in a plant promoter providing for synergic auxin inductiveness [43]. Here we found the AuxRE/AuxRE-like composite element consisting of AuxRE and its degenerative partner. The presence of this element in plant promoters significantly increase the feasibility for these genes to be involved in the plant developmental processes and to be auxin regulated, both was shown by functional annotation study (Additional file 12) and microarray data analysis (Additional file 8). We found that among AuxRE/AuxRE-like of the all possible structures, mainly those having direct AuxRE-like motif on the 3' flank of the direct AuxRE impact on auxin response (Additional file 12).

The AuxREs repeats can be the binding sites for ARF homodimers and heterodimers between different ARFs. They also may serve as the so called shadow enhancers. The shadow enhancers consisting of transcription factor binding site tandems, usually degenerative, were described as important cis-regulatory elements in a number of developmental genes in animals [44-46]. In the case of AuxRE/AuxRE-like composite elements, multiplication of weak sites into tandem clusters could make binding with ARF factors highly cooperative and strong.

## Conclusions

The auxin signaling mechanisms in plants are very diverse, engaging many transcription factors and their co-regulators. Deciphering auxin responsive elements code may reveal some basic features in ARF-Aux/IAA machinery and also disclose additional players in auxin signaling. Here we presented a comprehensive computational study on distribution and annotation of simple and composite auxin responsive elements in *A. thaliana*. We revealed some associations in location, orientation and composite structure of potential AuxREs with auxin response.

## Methods

### Data sets for the bioinformatic analysis

The following datasets were used for AuxRE context analysis and recognition:

1. *Training set*. 25 experimentally proven AuxRE sites (Additional file 1) were collected from published data [5,11,17,33,37,47-58]. Among them 19 were from *Arabidopsis thaliana*, 2 from *Pisum sativum L*., 2 from *Glycine max L*. and 1 from *Oryza sativa L*. and *Withania somnifera L.*. The sequences of the training set were aligned relative to TGTCNN with 50 nt flanks on both sides. The dataset was used to (1) deduce AuxRE_P&S_; (2) search motifs on the flanks of the core hexamer.

2. *Positive set *contained promoter sequences of 44 auxin responsive genes, taken from [18].

3. *Whole genome dataset *and genomic annotation data for *Arabidopsis thaliana *were taken from the Plant Ensembl database (MySQL server, http://www.ensembl.org/info/data/mysql.html)

4. Whole-genome multispecies alignment for *A. thaliana, A. lyrata, T. halophile, C. papaya and C. clementine *was downloaded from http://pipeline.lbl.gov/downloads.shtml of Vista Tools for comparative genomics [20].

### AuxRE recognition

The analysis of experimentally proven AuxREs set was done with two approaches: Optimized PWM (oPWM) and SiteGA [19]. The combination of both methods has been shown to be effective for large-scale genome transcription factors binding sites recognition [19]. Optimized PWM (oPWM) utilizes positional weight matrix for regulatory elements analysis and discovery. The procedure of matrix optimization, which implies the search of the best matrix location and length, defined the optimum length for AuxRE sequence as 25 nt with the core hexamer located in the center (Figure 1A-B, Additional file 1).

The second approach, SiteGA [19] used the same dataset for training. SiteGA is based on the genetic algorithm (GA) involving a discriminant function of locally positioned dinucleotides (LPD). The chosen set of LPD defines the SiteGA recognition function and allows computing the distribution of significant dependencies along the site sequence (Figure 1C) as follows. Initially we selected a significance level (e.g. p < 0.05). Next, we tested all the possible pairs of LPDs and compiled only those pairs that conformed significant positive or negative correlations, i.e. significant dependencies.

Potential AuxRE sites were predicted separately by both oPWM and SiteGA methods at specified stringencies. The thresholds 0.78/0.936 for recognition methods oPWM/SiteGA were chosen using the positive set, such as upstream regions of 32% of genes contain at least one potential AuxRE. If potential AuxRE passed these thresholds, we defined it as an AuxRE_P&S _variant.

The density of the site distribution was computed as the number of potential AuxREs per total number of tested positions in a sequence dataset. The abundance of potential AuxREs was estimated as the ratio of the AuxREs density in the analyzed region to that for the whole genome.

### AuxRE coupling motifs search

Motif search in the neighborhood of the experimentally proven AuxREs was done with the MotiGA algorithm, an implementation of genetic algorithm, based on computation of the p-value for a definite weight matrix score [59] and the KDIC measure for frequency matrix [60]. The description of MotiGA is given in Additional File 5[61]. As described above, 106 nt long sequences from the training set were used for the coupling elements search. To avoid an influence of AuxRE consensus, the hexamer core TGTCNN was masked. For each overrepresented motif, the MotiGA method computed matrix of the frequencies and weights. Each sequence from the training set was assigned by the *p*-values and the PWM score for the coupling motif. Finally, the threshold of PWM was chosen by a critical *p*-value for each motif separately (Additional file 3).

### Microarray data analysis

Sixteen microarray datasets on auxin treatment influences from published experiments (Table 2) were taken from the GEO database. The data were normalized using the RMA method and converted to log2 scale. The gene was considered as significantly up/down regulated by auxin, if the fold change between treatment and control was higher/lower than 1.5 and significant (*p *< 0.05) by the t-test (except GDS1408 which contained only one replica). Additionally, we required that at least 100 genes in each microarray were significantly up- or down-regulated. Associations between the presence of single or composite AuxRE in a gene promoter and its expression level were analyzed as follows. The portion of auxin up (down)-regulated genes observed in the experiment was compared with the respective portion observed for the subset of genes having a certain type of predicted AuxRE in a specified region. Namely, in this analysis we considered AuxREs recognized in the extended [-1500; 5'UTR] and proximal [-300; 5'UTR] regulatory regions. We considered an association as significant, if it was found at least in four microarray experiments (see below).

### Statistical analysis

To confirm statistical significance of the differences calculated for the gene subsets, we applied the following t-test for proportions. The proportions were computed as the ratio of *(a) *the number of auxin regulated genes (significantly up- or down-regulated genes, genes from the positive set) to *(b) *the total number of genes in the set. The first proportion *p_1 _*was computed for the set of genes, which had at least one potential AuxRE. The second proportion *p_2 _*was calculated for the whole genome. In microarray analysis, *p_2 _*was calculated for the genes which had a probe on the microchip. The angular (arcsine square-root) transformation *z(p) *was used to compute the t-test for two proportions, $z=2\text{arcsin}\left(\sqrt{p_{i}}\right)$[62].

The threshold for the number of microarray experiments whereas we found statistically significant association was set by the binomial trial estimate $P(N,k)=\sum_{k}^{N}C_{N}^{k}p^{k}{\left(1-p\right)}^{N-k}$. Namely, if we expect «success» in each of the sixteen tests with the probability *p *< 0.05, then we expect at least four «successes» in sixteen attempts with the probability *P*(16,4) < 0.01.

### Phylogenetic footprinting

Only continuous fragments (more than 100 nt in length) of alignment from Vista tool [20] conserved for four plant species were taken in analysis. A potential AuxRE site was considered as conserved if it was located to the existing alignment and its core sequence TGTC was conserved among the four plant species. To estimate an average conservation of the regulatory regions we mapped the extended promoter regions [-1500; 5'UTR] to the existing alignments. Among all 21098 genes analyzed in microarray data, we defined only 15060 with any conserved nt in the regulatory region.

### Functional annotation

Functional annotation was performed with the use of the AgriGO GO analysis toolkit [26]. For statistical analysis, we used the Fisher test method, for multiple hypothesis testing, we applied Hochberg FDR test [63] with a marginal p-value of 0.05. As input data we used a list of AGI codes of genes that contain certain elements in their regulatory regions. As the successful result, we took only those terms of functional annotation, which had undergone the mentioned above multiple testing.

## Abbreviations

AuxRE: Auxin Responsive Element; AuxRE_P&S_: AuxRE recognized in the present work by both oPWM and SiteGA methods; ARF: Auxin Response Factor; TSS: Transcription Start Site; ABRE: ABA-Responsive Element;

## Competing interests

The authors declare that they have no competing interests.

## Authors' contributions

Conceived and designed the study: VVM, NAO and VGL. Performed the calculations: DSW and VGL. Analyzed the data: VVM and NAO. Wrote the paper: VVM, NAO and VGL. Coordinated the project: VVM. All authors read and approved the final manuscript.

## Supplementary Material

Additional file 1 The training dataset of experimentally confirmed AuxREs used in the bioinformatical analysis. Click here for file

Additional file 2 The frequencies of the dinucleotides that were used to construct oPWM model for AuxRE prediction. x axis on the figure denotes the position relative to the centrally located AuxRE core sequence, indicated by the capital letters in the frame. Y axis marks the dinucleotide frequencies, i.e. counts of the specific dinucleotides in the certain position of the sequence alignment. Click here for file

Additional file 3 Description of Y-patch, AuxRE-like and ABRE-like coupling motifs, revealed by *de novo *motif search on the flanks of experimentally proven AuxREs. For each motif we compiled the frequency matrix with the estimated threshold; #seq denotes the number of the sequence in the Additional file 1; position, strand, PWM score, and sequences that passed the threshold. Logos constructed for these three sequence sets are shown on Figure 1D-F. Click here for file

Additional file 4 The significant correlations between the frequencies of the locally positioned dinucleotides (LPDs) for AuxREs from the *Training set *(Additional file 1). Correlations were deduced from the SiteGA model [19] for AuxRE recognition. Each horizontal strip depicts one correlation between two LPDs. A - positive correlations; B - negative correlations. The analyzed region consisted of 25 nt located [-12;+13] relative to the centrally positioned AuxRE core hexamer. Click here for file

Additional file 5 Supplementary methods. *De novo *search discovery by MotiGA. Click here for file

Additional file 6 The association of different AuxRE variants with auxin up-regulation. A. The portion of genes significantly up-regulated by auxin in more than three microarray experiments (Table 2) among the genes with a predicted AuxRE variant in [-1500; 5'UTR] regions. The basal level (dashed line) presents the average portion of auxin regulated genes in the whole genome. Namely, 1965 genes were significantly (>1,5 fold, p < 0,05) up-regulated in more than three microarrays (Table 1) among 21098 genes, which were detected by the microarray platform. B. The portion of up-regulated by auxin genes with conservative AuxRE among the up-regulated genes having an AuxRE. The plot shows that AuxRE_P&S _related to auxin up-regulation are more conservative than other AuxRE variants. The basal conservation level denotes the portion of up-regulated genes which have any conservative nucleotide in the alignment of [-1500; 5'UTR] regions. Namely, 1442 out of 1965 genes. Statistics was calculated by t-test for proportions, * - *p *< 0.05; ** - *p *< 0.01. Click here for file

Additional file 7 Supplementary Table containing predictions for composite AuxRE elements in *A. thaliana *regulatory regions [-1500; 5'UTR]. Click here for file

Additional file 8 The summary table on microarray data analysis for the genes having in their regulatory regions predicted AuxRE variants. The portion of auxin up/down-regulated genes observed in the microarray was compared with the respective portion observed for the subset of genes having the certain variant of predicted AuxRE in a specified region. Significant increase in the portion by *t*-test for proportions in at least three microarrays was considered as significant association (see Methods for details). The specified regions are: [-1500; +5'UTR], [-300; +5'UTR]. The AuxRE variants are: (1) single/multiple; (2) predicted by SiteGA, oPWM, or by both methods (P&S), by TGTCTC or TGTSTSBC consensuses; (3) located in direct (+) or reverse (-) strand; (4) simple or composite; (5) composite AuxREs with a certain location of Y-Patch, ABRE-like or AuxRE-like relative to predicted AuxRE. Click here for file

Additional file 9 The analysis of ABRE-like motif frequency matrix by the motif comparison tool TOMTOM [23]. The search revealed the best match to MA0128.1 matrix for EmBP-1 (*p *< 0.005) is shown on the left, two logos on the right correspond to MA0128.1 and ABRE-like motif (HDSDYKS, see Additional file 3 for the frequency matrix). Click here for file

Additional file 10 Distribution of potential composite AuxRE relative to TSS (position +1) with respect to relative orientation of the coupling motifs. A. AuxRE/AuxRE-like. B. AuxRE/ABRE-like. Click here for file

Additional file 11 Functional annotation of the genes with predicted composite element AuxRE+/ ABRE-like in their regulatory [-1500; 5'UTR] region. Functional annotation was performed using the singular enrichment analysis from AgriGO analysis tool [26] under the Hochberg FDR multitest adjustment method. Each node in the graph represents a definite GO term with the value of FDR in the brackets. The yellow nodes represent significantly enriched terms, p < 0.002 (see annotation of the Figure 3). Click here for file

Additional file 12 Functional annotation of the genes with predicted composite element AuxRE+/AuxRE-like+ in their regulatory [-1500; 5'UTR] region. Functional annotation was performed using the singular enrichment analysis from AgriGO analysis tool [26] under the Hochberg FDR multitest adjustment method. Each node in the graph represents a definite GO term with the value of FDR in the brackets. The yellow nodes represent significantly enriched terms, p < 0.002 (see annotation of the Figure 3). Click here for file

## Declarations

Publication of this article has been funded by the Russian Science Foundation.

This article has been published as part of *BMC Genomics *Volume 15 Supplement 12, 2014: Selected articles from the IX International Conference on the Bioinformatics of Genome Regulation and Structure\Systems Biology (BGRS\SB-2014): Genomics. The full contents of the supplement are available online at http://www.biomedcentral.com/bmcgenomics/supplements/15/S12.
