# Supplementary material for: Computational analysis of auxin responsive elements in the Arabidopsis thaliana L. genome
Source: BMC Genomics. 2014 Dec 19;15(Suppl 12):S4. doi: 10.1186/1471-2164-15-S12-S4 (PMC4331925; doi:10.1186/1471-2164-15-S12-S4)
Supplement: Additional file 1 — The training dataset of experimentally confirmed AuxREs used in the bioinformatical analysis. [file 1471-2164-15-S12-S4-S1.docx]

**Additional file 1.** The training dataset of experimentally confirmed AuxRE sites used in the bioinformatical analysis.

| #seq | Sequence* | Description** | TSS, CRS, Strand*** | | | References |
| --- | --- | --- | --- | --- | --- | --- |
| 1 | caaaggcaagctaagttgtcggtagcaacatcaacagattt aaagaatggTGTCTCcttatagggg tgacaagaatgggacatattcacctaccatttggtctgga | *Pisum sativum*, Ps-IAA4/5 | -159 | -254 | - | [1] |
| 2 | ctcaaccatccaaattccagaccaaatggtaggtgaatatg tcccattctTGTCACccctataagg agacaccattctttaaatctgttgatgttgctaccgacaa | *Pisum sativum*, Ps-IAA4/5 | -173 | -268 | + |  |
| 3 | attaaaacccaataaattagaagtctcatgttgcagtctcc attattgatTGTCTCcttagaatcc aacatgtgaccctccattgtctaccattcacaccctttgg | AT2G42430 (LBD16) | -762 | -986 | + | [2] |
| 4 | atatttctgatattttcgtatcatttaaaatgaaaatttca tcattattcTGTCTCaaactacaac atcttgatggtaaaaaattttatcgcatagcagttgtgga | AT3G58190 (LBD29) | -612 | -672 | - |  |
| 5 | cctgaccctacgtactgttcaaatatatcatataccagacg tttgtccatTGTCTCtatatatcgt tggcacattttcttttacatgttagaaatataagttaaag | At3G58190 (LBD29) | -1373 | -1443 | + |  |
| 6 | gacacatctggacccacatgtcggccaccatgcaccatccc tggccctcgTGTCTCctcaataagc tacacaatttgaaacatacacgcaatcctttgtctcaata | Glycine max, X60033 (GH3-D1) | -173 | -330 | + | [3] |
| 7 | ctcgtgtctcctcaataagctacacaatttgaaacatacac gcaatccttTGTCTCaataagttcc actcaggtactgttttctcccgcaaccatgacgtaattct | Glycine max ,X60033 (GH3-D4) | -127 | -284 | + |  |
| 8 | tttgcttgtaaaattacagaattaacgcaggggttttaatt gctgcttatTGTCTCattcagtcat ttgccgaccaaccaacataaatctcaatataatgagttca | AT1G12980 (DRN/ESR1) | -1105 | -1178 | + | [4] |
| 9 | gacagaagcaataaataaaaaacaagcttctgatacatgaa atacatataTGTCTCatacatacgt ttagacaaacctgaaatgtcctcttcgtacaataatatcc | AT1G12980 (DRN/ESR1) | -720 | -793 | + |  |
| 10 | ggagagaaatggagaaggattagggccaggggattaaaaaa aatcgatctTGTCTCcccaatgggc aaccagctccattattttgagctcttctccttctttgttt | *Oryza sativa*, AAN87738 (CRL1) |  | -173 | - | [5] |
| 11 | taaattaatcattgtctttgcttcaaagccaatcctatgag actttgtctTGTCTCcaacttgtat ataagctctattcctctactctgtttcacaccatctcttc | AT4G35350 (XCP1) | -42 | -88 | + | [6] |
| 12 | tgaattaaaatgaagaaaaaaaagaatgagaaaaagtgaga agtggtggtTGTCTGgtattaaggg tactcacttctctccttttcaacacagcccgacacacatg | AT4G32880 (ATHB8) | -143 | -946 | + | [7] |
| 13 | gttaaacattctctttcctccacgtatatatatatactctt cgtaactctTGTCTCtcttctcttc gtctagttaaaactctcggagctcctttcagttctcgggt | AT1G31880 (BRX, site A) | -13 | -2327 | + | [8] |
| 14 | tcccttggcctgtcaaatgtatcgtgaccaaaacccgaccc ggtttcagcTGTCTCaaaccaatca gaaaccgccacgtattctacttccaccacacgtaagccat | AT1G31880 (BRX, site B) | -246 | -2560 | + |  |
| 15 | atctaaaggcgatgagtgatgtgtttatgtcttgtcagaaa acgtaatatTGTGTCttcacggtca taatctttttagggacccaattttctttgattttacgatc | AT1G74900 (ARR15) |  | -896 | - | [9] |
| 16 | cacatggctttgggttgtagatactaaaccaagaacaaatc aataaatggTGTCTGagaagttagt gtctaatgatgtcctacatgataacttcattggggcttat | AT4G38850 (SAUR15, site A2) | -146 | -253 | - | [10] |
| 17 | agttgaatcaaaagtgaagtctctctatcttctctctctat caagctcatTGTCTCtctatttata acaacacttcacttccattctcaaacagcaccatcatctt | AT5G18560 (PUCHI) | -68 | -154 | + | [11] |
| 18 | cggtcataatctttttagggacccaattttctttgatttta cgatcctttTGTCTGctctttcttt ctatctgacaatgactactctcctctctttttatctttcg | AT1G74900 (ARR15 box A) |  | -836 | - | [9] |
| 19 | ccacatgtctgcccaaaactagccaaagattacgtgaccgc ggtccctctTGTCCCctgtctcggt ctaacgataacaaaccgagcccacttttatgtcgacgtgg | AT2G23170 (GH3.3) | -118 | -180 | + | [12] |
| 20 | gtgtaaattaattgtaatctatgttgcatccgatgctagct atataatgtTGTCTGtagaatcaag tttctaaaatgttcaaaaggaaaagttagaaaaatatcta | AT5G13790 (AGL15) | -1143 | -1185 | + | [13] |
| 21 | ctatatatacacatgtgtatgcattatcaaatgggatgttt gtgacctttTGTCTCttcttctcta aaattctctttctcccaaaaactaaaaaaccaaaaaaacc | AT3G25710 (TMO5) | +22 | -59 | + | [14] |
| 22 | cctaaactccataacctgtttcaccgataaagtgcctttgc ttctatctcTGTCACtcttactact tgttgaacaatattctacaaaaaaatgtcgggaagaagat | AT1G74500 (TMO7) | +116 | -37 | + |  |
| 23 | caccgttcccggatattactaaccttgaagtggcttgttcc ccggagcacTGTCTCttccctgtca atttctataaaaataacaataatcttccttttttcttgtt | AT5G19040 (IPT5) | -141 | -174 | - | [15] |
| 24 | actctcacttgtatctgaaagaaaaactaaaaataaagtgg aggagagatTGTCTCtatagctttt tgcaaaagagaaacaaaaaaaaaaacgaacgtacgtgtga | AT4G39400 (BRI1) | -981 | -1147 | - | [16] |
| 25 | agacaacatagctatagtttcatagactttcttcaaacaag agaacctcgTGTCCCacacttaact acacttaactacctgtttgttttaaaaagttaaaatgact | *Withania_somnifera*, FM956482 (a206) | +1195 | +876 | + | [17] |

* the blanks mark the central fragments of AuxRE 25nt in length, which we used to train oPWM & SiteGA recognition models; the caps letters denote the AuxRE core;

** gene name (AGI for *A.thailana,* for other species the name and EMBL AC);

*** positions relative to transcription start site (TSS) and coding region start site (CRS), direct (+) or reverse (-) orientations; the absence of position relative to TSS denotes that only CRS position is known for a gene.

**References**

1. Ballas N, Wong LM, Ke M, Theologis A: **Two auxin-responsive domains interact positively to induce expression of the early indoleacetic acid-inducible gene PS-IAA4/5.** *Proc Natl Acad Sci* 1995, **92**:3483–3487.

2. Okushima Y, Mitina I, Quach HL, Theologis A: **AUXIN RESPONSE FACTOR 2 (ARF2): a pleiotropic developmental regulator.** *Plant J* 2005, **43**:29–46.

3. Ulmasov T, Liu ZB, Hagen G, Guilfoyle TJ: **Composite structure of auxin response elements.** *Plant Cell* 1995, **7**:1611–1623.

4. Cole M, Chandler J, Weijers D, Jacobs B, Comelli P, Werr W: **DORNROSCHEN is a direct target of the auxin response factor MONOPTEROS in the Arabidopsis embryo.** *Development* 2009, **136**:1643–1651.

5. Inukai Y, Sakamoto T, Ueguchi-tanaka M, Shibata Y, Gomi K, Umemura I, Hasegawa Y, Ashikari M, Kitano H, Matsuoka M: **Crown rootless1 , Which Is Essential for Crown Root Formation in Rice , Is a Target of an AUXIN RESPONSE FACTOR in Auxin Signaling**. *Plant Cell* 2005, **17**:1387–1396.

6. Ismail IO: **Function and Regulation of Xylem Cysteine Protease 1 and Xylem Cysteine Protease 2 in Arabidopsis. Dissertation.** Virginia Polytechnic Institute; 2004.

7. Donner TJ, Sherr I, Scarpella E: **Regulation of preprocambial cell state acquisition by auxin signaling in Arabidopsis leaves.** *Development* 2009, **136**:3235–3246.

8. Scacchi E, Salinas P, Gujas B, Santuari L, Krogan N, Ragni L, Berleth T, Hardtke CS: **Spatio-temporal sequence of cross-regulatory events in root meristem growth.** *Proc Natl Acad Sci* 2010, **107**:22734–22739.

9. Zhao Z, Andersen SU, Ljung K, Dolezal K, Miotk A, Schultheiss SJ, Lohmann JU: **Hormonal control of the shoot stem-cell niche**. *Nature* 2010, **465**:1089–1092.

10. Walcher CL, Nemhauser JL: **Bipartite promoter element required for auxin response.** *Plant Physiol* 2012, **158**:273–282.

11. Hirota A, Kato T, Fukaki H, Aida M, Tasaka M: **The auxin-regulated AP2/EREBP gene PUCHI is required for morphogenesis in the early lateral root primordium of Arabidopsis.** *Plant Cell* 2007, **19**:2156–2168.

12. Berendzen KW, Weiste C, Wanke D, Kilian J, Harter K, Dröge-Laser W: **Bioinformatic cis-element analyses performed in Arabidopsis and rice disclose bZIP- and MYB-related binding sites as potential AuxRE-coupling elements in auxin-mediated transcription.** *BMC Plant Biol* 2012, **12**:125.

13. Zhu C, Perry SE: **Control of expression and autoregulation of AGL15, a member of the MADS-box family.** *Plant J* 2005, **41**:583–594.

14. Schlereth A, Möller B, Liu W, Kientz M, Flipse J, Rademacher EH, Schmid M, Jürgens G, Weijers D, Mo B, Ju G: **MONOPTEROS controls embryonic root initiation by regulating a mobile transcription factor.** *Nature* 2010, **464**:913–916.

15. Cheng ZJ, Wang L, Sun W, Zhang Y, Zhou C, Su YH, Li W, Sun TT, Zhao XY, Li XG, Cheng Y, Zhao Y, Xie Q, Zhang XS: **Pattern of auxin and cytokinin responses for shoot meristem induction results from the regulation of cytokinin biosynthesis by AUXIN RESPONSE FACTOR3.** *Plant Physiol* 2013, **161**:240–251.

16. Sakamoto T, Fujioka S: **Auxins increase expression of the brassinosteroid receptor and brassinosteroid-responsive genes in Arabidopsis.** *Plant Signal Behav* 2013, **8**:e23509.

17. Khan MR, Hu J, Ali GM: **Reciprocal loss of CArG-boxes and auxin response elements drives expression divergence of MPF2-Like MADS-box genes controlling calyx inflation.** *PLoS One* 2012, **7**:e42781.
