# Supplementary figures and images for: Computational analysis of auxin responsive elements in the Arabidopsis thaliana L. genome
Source: BMC Genomics. 2014 Dec 19;15(Suppl 12):S4. doi: 10.1186/1471-2164-15-S12-S4 (PMC4331925; doi:10.1186/1471-2164-15-S12-S4)

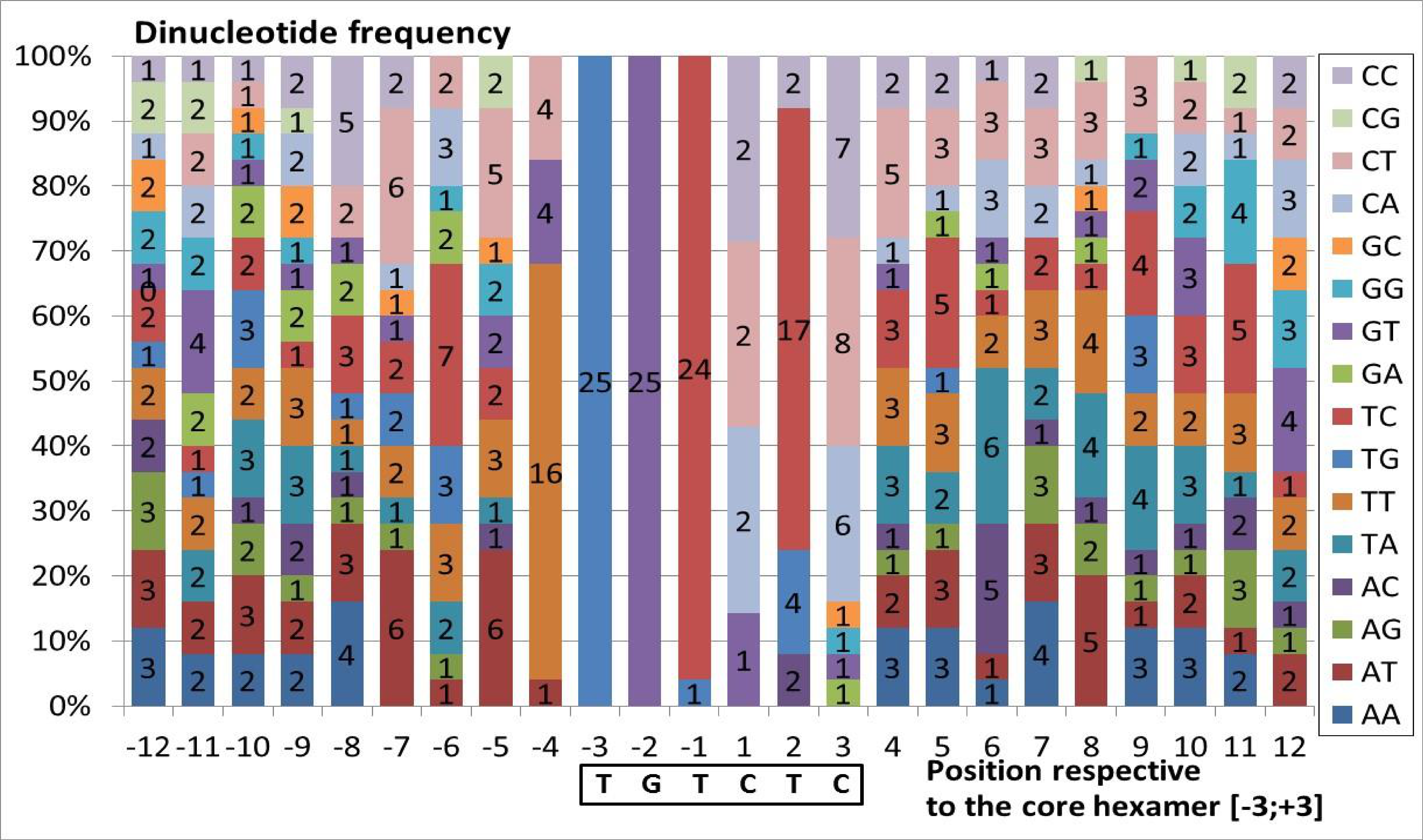

Supplement: Additional file 2 — The frequencies of the dinucleotides that were used to construct oPWM model for AuxRE prediction. x axis on the figure denotes the position relative to the centrally located AuxRE core sequence, indicated by the capital letters in the frame. Y axis marks the dinucleotide frequencies, i.e. counts of the specific dinucleotides in the certain position of the sequence alignment. [file 1471-2164-15-S12-S4-S3.tif]

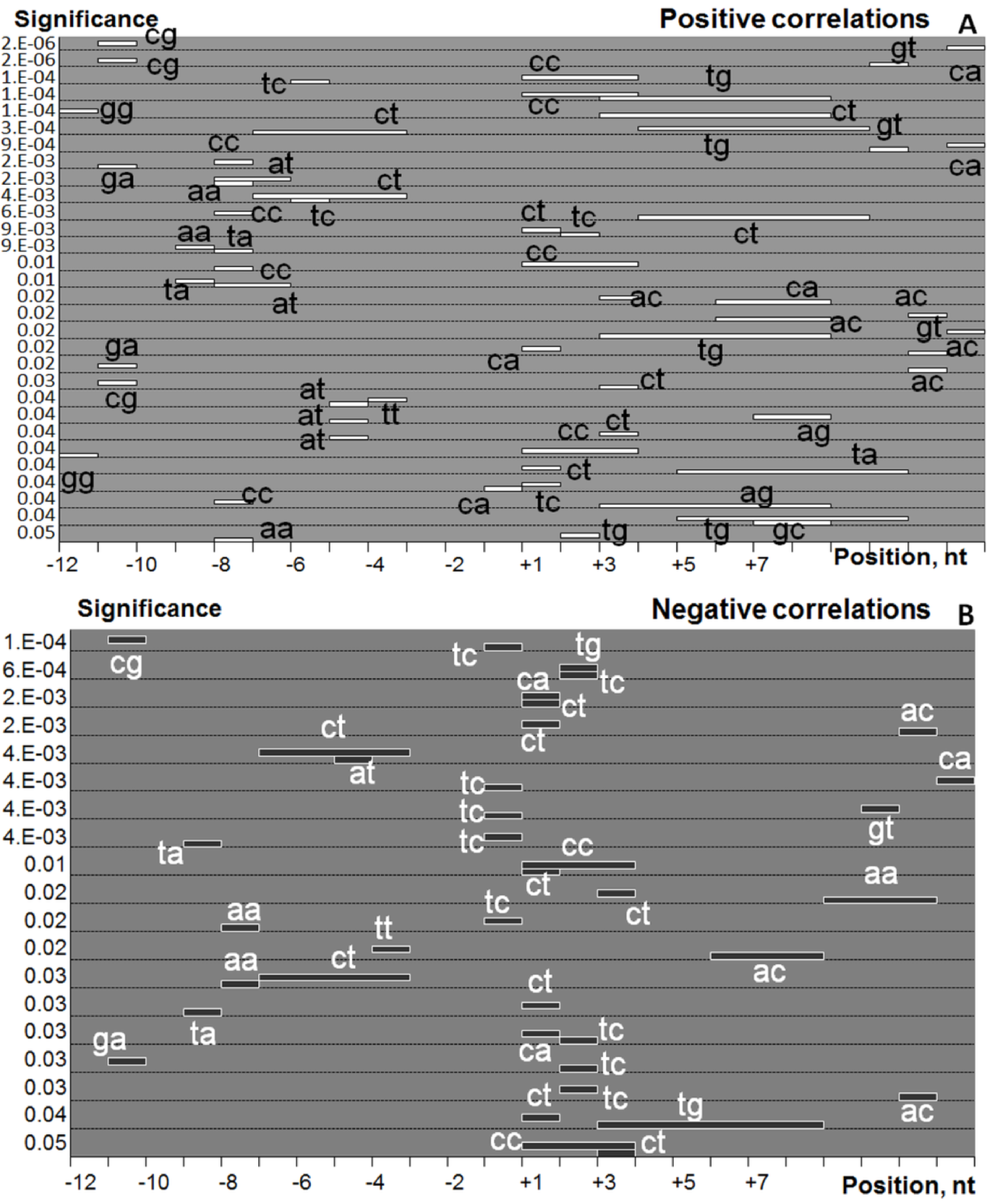

Supplement: Additional file 4 — The significant correlations between the frequencies of the locally positioned dinucleotides (LPDs) for AuxREs from the Training set (Additional file 1). Correlations were deduced from the SiteGA model [19] for AuxRE recognition. Each horizontal strip depicts one correlation between two LPDs. A - positive correlations; B - negative correlations. The analyzed region consisted of 25 nt located [-12;+13] relative to the centrally positioned AuxRE core hexamer. [file 1471-2164-15-S12-S4-S4.tif]

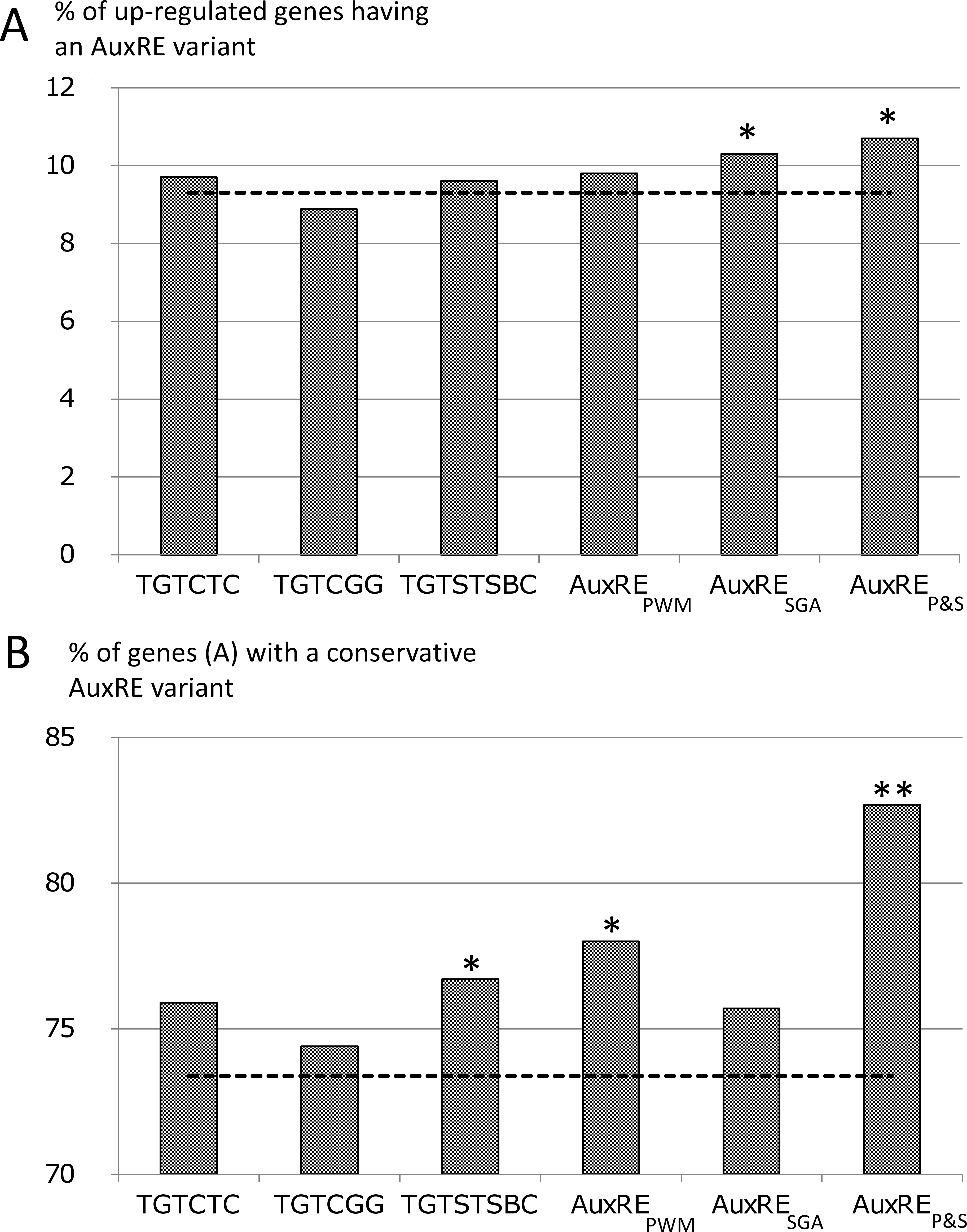

Supplement: Additional file 6 — The association of different AuxRE variants with auxin up-regulation. A. The portion of genes significantly up-regulated by auxin in more than three microarray experiments (Table 2) among the genes with a predicted AuxRE variant in [-1500; 5'UTR] regions. The basal level (dashed line) presents the average portion of auxin regulated genes in the whole genome. Namely, 1965 genes were significantly (>1,5 fold, p < 0,05) up-regulated in more than three microarrays (Table 1) among 21098 genes, which were detected by the microarray platform. B. The portion of up-regulated by auxin genes with conservative AuxRE among the up-regulated genes having an AuxRE. The plot shows that AuxREP&S related to auxin up-regulation are more conservative than other AuxRE variants. The basal conservation level denotes the portion of up-regulated genes which have any conservative nucleotide in the alignment of [-1500; 5'UTR] regions. Namely, 1442 out of 1965 genes. Statistics was calculated by t-test for proportions, * - p < 0.05; ** - p < 0.01. [file 1471-2164-15-S12-S4-S7.tif]

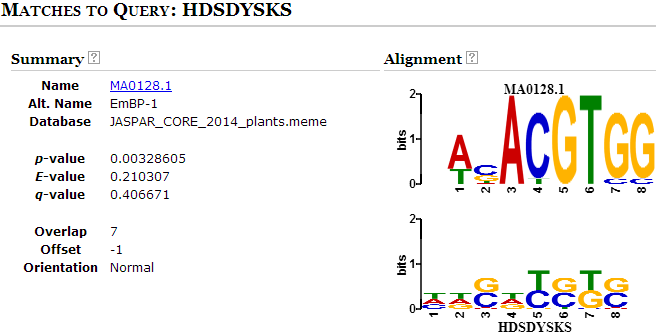

Supplement: Additional file 9 — The analysis of ABRE-like motif frequency matrix by the motif comparison tool TOMTOM [23]. The search revealed the best match to MA0128.1 matrix for EmBP-1 (p < 0.005) is shown on the left, two logos on the right correspond to MA0128.1 and ABRE-like motif (HDSDYKS, see Additional file 3 for the frequency matrix). [file 1471-2164-15-S12-S4-S5.tif]

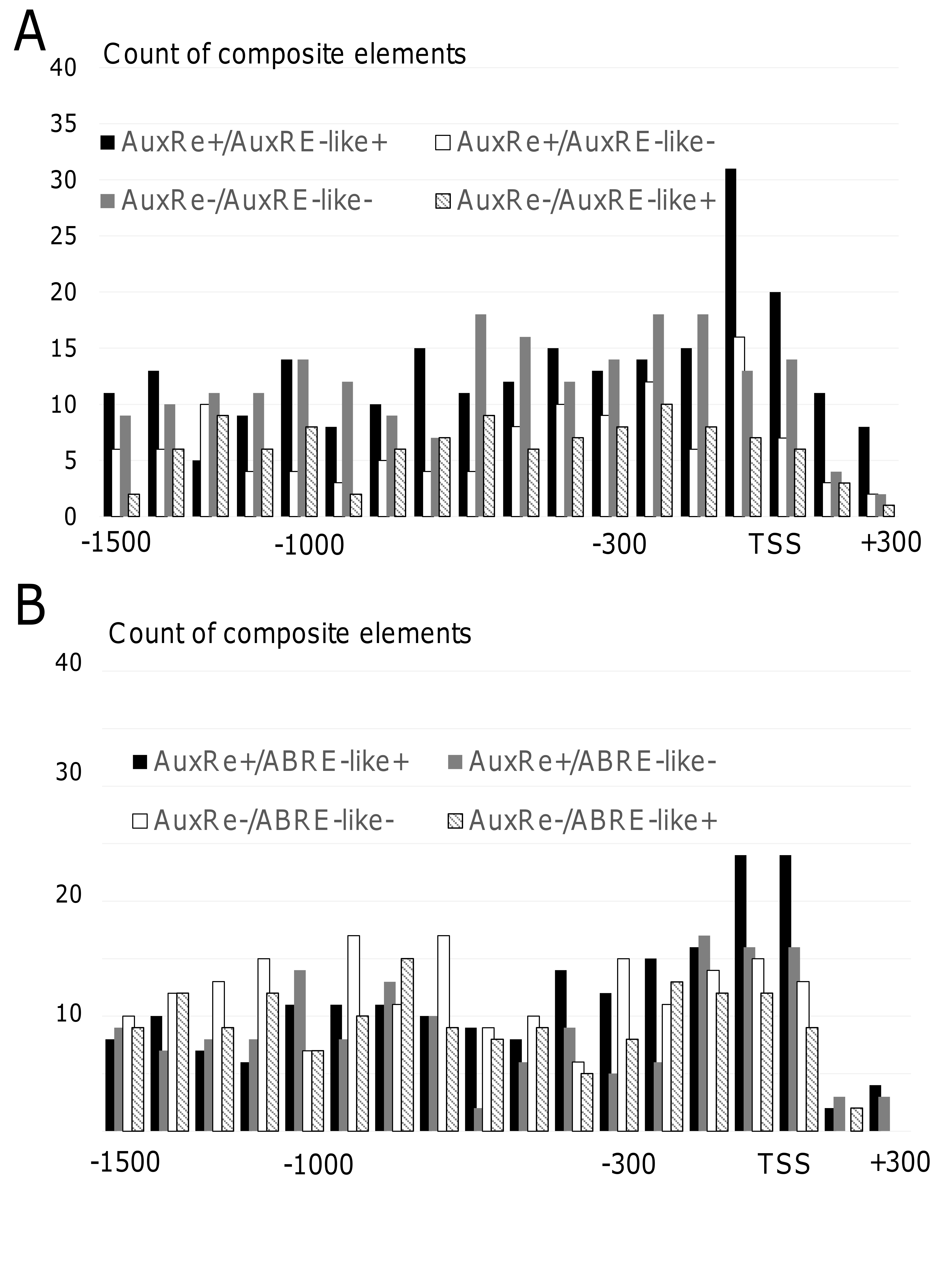

Supplement: Additional file 10 — Distribution of potential composite AuxRE relative to TSS (position +1) with respect to relative orientation of the coupling motifs. A. AuxRE/AuxRE-like. B. AuxRE/ABRE-like. [file 1471-2164-15-S12-S4-S10.tif]

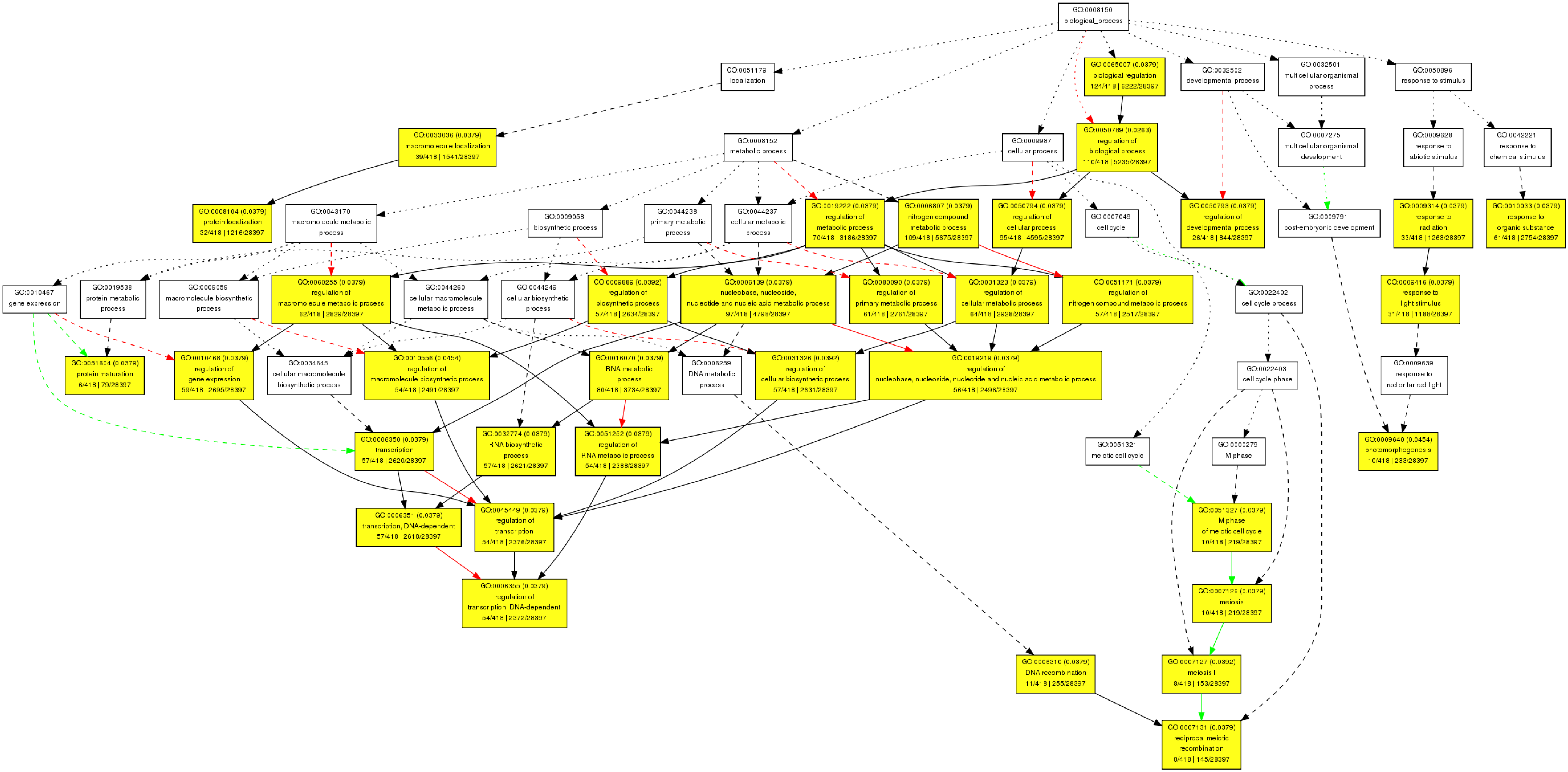

Supplement: Additional file 11 — Functional annotation of the genes with predicted composite element AuxRE+/ ABRE-like in their regulatory [-1500; 5'UTR] region. Functional annotation was performed using the singular enrichment analysis from AgriGO analysis tool [26] under the Hochberg FDR multitest adjustment method. Each node in the graph represents a definite GO term with the value of FDR in the brackets. The yellow nodes represent significantly enriched terms, p < 0.002 (see annotation of the Figure 3). [file 1471-2164-15-S12-S4-S11.tif]

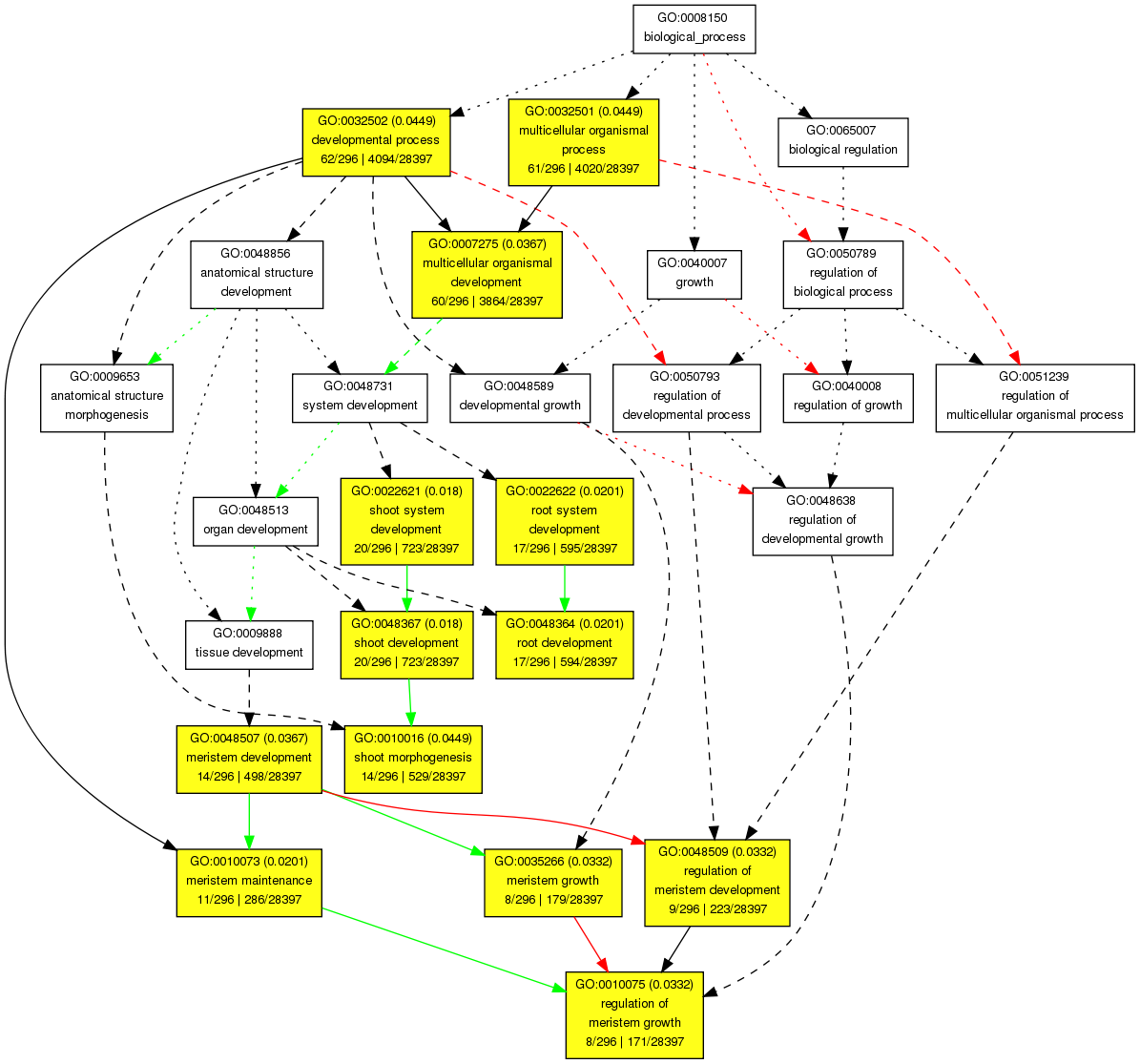

Supplement: Additional file 12 — Functional annotation of the genes with predicted composite element AuxRE+/AuxRE-like+ in their regulatory [-1500; 5'UTR] region. Functional annotation was performed using the singular enrichment analysis from AgriGO analysis tool [26] under the Hochberg FDR multitest adjustment method. Each node in the graph represents a definite GO term with the value of FDR in the brackets. The yellow nodes represent significantly enriched terms, p < 0.002 (see annotation of the Figure 3). [file 1471-2164-15-S12-S4-S12.tif]
