# Supplementary material for: Computational analysis of auxin responsive elements in the Arabidopsis thaliana L. genome
Source: BMC Genomics. 2014 Dec 19;15(Suppl 12):S4. doi: 10.1186/1471-2164-15-S12-S4 (PMC4331925; doi:10.1186/1471-2164-15-S12-S4)
Supplement: Additional file 3 — Description of Y-patch, AuxRE-like and ABRE-like coupling motifs, revealed by de novo motif search on the flanks of experimentally proven AuxREs. For each motif we compiled the frequency matrix with the estimated threshold; #seq denotes the number of the sequence in the Additional file 1; position, strand, PWM score, and sequences that passed the threshold. Logos constructed for these three sequence sets are shown on Figure 1D-F. [file 1471-2164-15-S12-S4-S2.docx]

**Additional File 2**. Description of Y-patch, AuxRE-like and ABRE-like coupling motifs, revealed by *de novo* motif search on the flanks of experimentally proven AuxREs. For each motif we compiled the frequency matrix with the estimated threshold; #seq denotes the number of the sequence in the Additional file 1; position, strand, PWM score, and sequences that passed the threshold. Logos constructed for these three sequence sets are shown on Figure 1D-F.

| Name of motif | Frequency matrix and threshold for the respective PWM | #seq | Position, strand | PWM score | Sequence |
| --- | --- | --- | --- | --- | --- |
| Y-patch | A 0 0 0 1 0 0 0 1  C 0 25 0 22 25 0 12 7  G 0 0 0 0 0 0 0 0  T 25 0 25 2 0 25 13 17  0.91 | 18 | 89+ | 1 | TCTCCTCT |
|  |  | 12 | 81+ | 0.9957 | TCTCCTTT |
|  |  | 24 | 43- | 0.9825 | TCTCCTCC |
|  |  | 10 | 96+ | 0.9782 | TCTCCTTC |
|  |  | 13 | 61+ | 0.9301 | TCTTCTCT |
|  |  | 17 | 32+ | 0.9301 | TCTTCTCT |
|  |  | 21 | 62+ | 0.9301 | TCTTCTCT |
|  |  | 7 | 11+ | 0.9274 | TCTCCTCA |
|  |  | 2 | 65- | 0.9231 | TCTCCTTA |
|  |  | 11 | 85+ | 0.9139 | TCTACTCT |
| AuxRE-like | A 9 0 0 2 0 0 0 9  C 0 0 0 7 0 25 0 7  G 7 0 0 7 0 0 0 0  T 9 25 25 9 25 0 25 9  0.9997 | 5 | 77+ | 1 | ATTTTCTT |
|  |  | 12 | 16- | 1 | TTTTTCTT |
|  |  | 15 | 90+ | 1 | ATTTTCTT |
|  |  | 18 | 30+ | 1 | ATTTTCTT |
|  |  | 20 | 96- | 1 | TTTTTCTA |
|  |  | 23 | 100+ | 1 | TTTTTCTT |
|  |  | 24 | 22- | 1 | TTTTTCTT |
|  |  | 3 | 86+ | 0.9997 | ATTGTCTA |
|  |  | 9 | 71- | 0.9997 | TTTGTCTA |
|  |  | 11 | 15+ | 0.9997 | ATTGTCTT |
|  |  | 13 | 12+ | 0.9997 | ATTCTCTT |
|  |  | 21 | 73+ | 0.9997 | ATTCTCTT |
| ABRE-like | A 9 9 1 9 0 0 0 1  C 7 0 12 0 12 12 0 12  G 0 7 12 7 0 12 12 12  T 9 9 0 9 13 1 13 0  0.9949 | 6 | 22+ | 1 | ATGTCGGC |
|  |  | 13 | 102+ | 1 | TTCTCGGG |
|  |  | 22 | 95+ | 1 | ATGTCGGG |
|  |  | 8 | 72- | 0.9997 | TGGTCGGC |
|  |  | 23 | 45- | 0.9997 | TGCTCCGG |
|  |  | 9 | 86+ | 0.9949 | ATGTCCTC |
