# Supplementary material for: Computational analysis of auxin responsive elements in the Arabidopsis thaliana L. genome
Source: BMC Genomics. 2014 Dec 19;15(Suppl 12):S4. doi: 10.1186/1471-2164-15-S12-S4 (PMC4331925; doi:10.1186/1471-2164-15-S12-S4)
Supplement: Additional file 5 — Supplementary methods. De novo search discovery by MotiGA. [file 1471-2164-15-S12-S4-S6.docx]

**Supplementary Methods**

***De novo* search discovery by MotiGA**

We employed a genetic algorithm (GA) based method MotiGA similar to previous implementation SiteGA [Levitsky et al., 2007] to search for motifs represented as a PWM/PFM. The GA taken the input data as the dataset *Ω* of *N* nucleotide sequences {*S_1_…S_n_…S_N_*} and the fixed length *k* of motif was the input parameter. The dataset *Ω* was described by nucleotide frequencies (*p_a_, p_t_, p_g_, p_c_*). A motif *ξ* was represented by related matrices of frequencies {*f_i,j_*} and weights {*w_i,j_*}. Both matrices had the size 4×L. For any *i*-th column of frequency matrix we required that $\sum_{j=1}^{4} f_{ij}=N$, since the motif compiled one *k*-mer from each sequence of the dataset. We compute weights *w_i,j_* as follow: $Ln\left[\frac{f_{i,j}+0.25}{N+1}/p_{j}\right]$ [Wasserman and Sandelin, 2004; Levitsky et al., 2007]. Matrix score for any *k-*mer *X_1_X_2_…X_k_* was computed as the sum of weights for respective nucleotide types and positions: $\sum_{i=1}^{k} w_{i,j}$. The matrix score was normalised to the interval [0; 1] [Levitsky et al., 2007].

The GA optimized the set (population) of motifs (individuals), so that fitness function *ψ(ξ)* for any motif was maximized. This function *ψ(ξ)* was calculated as ratio *T(ξ)/F(ξ)* of estimate for the motif content for the dataset *Ω* to that expected on the basis of nucleotide content of this dataset. Namely, the value *T(ξ)* we computed as the Kullback-Leibler Discreate Information Content [KDIC, Kulakovskiy *et al.,* 2010] as follow: $KDIC=\sum_{i=1}^{k} KDIC(i)$, here$KDIC(i)=\frac{1}{N}\left[\sum_{acgt} Log\left[f_{i,j}!\right]-Log\left[N!\right]\right]-\sum_{acgt} \left[\frac{f_{i,j}}{N}Log\left[p_{j}\right]\right]$. This measure reflected the column conservation in the frequency matrix {*f_i,j_*}.

To evaluate *F(ξ)*: (a) application of PWM {*w_i,j_*} provided the best scoring *k*-mers {*σ_n_*} for each sequence *S_n_* the dataset Ω; (b) respective the best scores {*BS(σ_1_)...BS(σ_N_*)} of matrix {*w_ij_*} were computed. Than for each score *BS(σ_n_)* p-value *PV(σ_N_)* was computed as follow. The p-value for score *S(σ*) was defined as the fraction of the total dictionary (all sequences of length *k*) that had scores equal or greater than *S(σ*). For example, for the length *k* the dictionary size is *4^k^*. If among them only Q sequences have scores equal or greater than *S(σ*), than *p-*value is equal to *Q/4^k^* . The algorithm [Touzet and Varre, 2007] was applied to compute the dependence of p-value from matrix score for a given matrix. Finally *F(ξ)* value was estimated as .

GA started from the population of *P* arbitrary assigned motifs *{ξ_1_, ξ_2_,…, ξ_P_}.* Genetic operators mutation and recombination were defined as a shift in nucleotide distribution in a column of frequency matrix {*f_ij_*} of a motif *ξ* and an exchange of respective columns between two distinct motifs *ξ_1_* and *ξ_2_*. Application of these operators gradually moved the population to the local maxima of the fitness function. This maximization implied an overrepresentation of high-scoring motifs in the dataset *Ω* in the comparison with the expectation based on nucleotide content.

Kulakovskiy IV, Boeva VA, Favorov AV, Makeev VJ. (2010) Deep and wide digging for binding motifs in ChIP-Seq data. *Bioinformatics*, 26(20):2622-2623.

Levitsky VG, Ignatieva EV, Ananko EA, Turnaev II, Merkulova TI, Kolchanov NA, Hodgman TC (2007) Effective transcription factor binding site prediction using a combination of optimization, a genetic algorithm and discriminant analysis to capture distant interactions. *BMC Bioinformatics,* 8:481.

Touzet H and Varre JS. (2007) Efficient and accurate P-value computation for Position Weight Matrices. *Algorithms for Molecular Biology,* 2:15.

Wasserman WW, Sandelin A (2004) Applied bioinformatics for the identification of regulatory elements. *Nat Rev Genet* 2004, 5(4):276-287.
